# Changing landscape of steatotic liver diseases and liver fibrosis in the United States during the COVID-19 pandemic

**DOI:** 10.1097/HC9.0000000000000806

**Published:** 2025-09-05

**Authors:** Abdelrahman M. Attia, Mohammad Saeid Rezaee-Zavareh, Yee Hui Yeo, Minsun Kwak, Hyunseok Kim, Mazen Noureddin, Ju Dong Yang

**Affiliations:** 1 Department of Internal Medicine, Karsh Division of Gastroenterology and Hepatology, Cedars-Sinai Medical Center, Los Angeles, California, USA; 2 Department of Internal Medicine, Comprehensive Transplant Center, Cedars-Sinai Medical Center, Los Angeles, California, USA; 3 Middle East Liver Diseases (MELD) Center, Tehran, Iran; 4 Department of Internal Medicine, Healthcare Research Institute, Healthcare System Gangnam Center, Seoul National University Hospital, Seoul, Republic of Korea; 5 Department of Houston Research Institute and Houston Methodist Hospital, Houston, Texas, USA; 6 Samuel Oschin Comprehensive Cancer Institute, Cedars-Sinai Medical Center, Los Angeles, California, USA

**Keywords:** ALD, MASLD, MetALD, NHANES, steatotic liver diseases

## Abstract

**Background:**

Steatotic liver diseases (SLDs) and their subcategories—metabolic dysfunction–associated steatotic liver disease (MASLD), metabolic dysfunction and alcohol-associated liver disease (MetALD), and alcohol-associated liver disease (ALD)—significantly contribute to liver-related and extrahepatic morbidity and mortality. This project aimed to assess the landscape of SLDs and clinically significant fibrosis (CSF) before (2017–2020) and during (2021–2023) the COVID-19 pandemic.

**Methods:**

Using National Health and Nutrition Examination Survey (NHANES) data, we analyzed 8965 prepandemic and 6337 pandemic participants aged ≥18 years. The main evaluated outcomes were changes in age-adjusted mean CSF, mean controlled attenuation parameter score, and age-adjusted prevalence of MASLD, MetALD, and ALD before and during the pandemic.

**Results:**

The age-adjusted prevalence of SLDs changed significantly (*p*=0.003) between the prepandemic and pandemic periods. ALD prevalence rose from 0.94% to 2.27%, MetALD from 2.60% to 4.42%, while MASLD declined from 30.13% to 25.46%. Vigorous and moderate physical activity decreased significantly (*p*<0.001), whereas moderate/excessive alcohol intake increased (*p*<0.001). The prevalence of CSF increased from 8.3% to 10.5% (*p*=0.028). Multivariable analyses showed the COVID-19 pandemic (adjusted OR: 1.47, 95% CI: 1.00–2.17) and moderate/excessive alcohol intake (adjusted OR: 2.13, 95% CI: 1.15–3.95) were associated with CSF. In addition, older age, higher body mass index, larger waist circumference, prediabetes/diabetes, and lower income were each independently associated with CSF.

**Conclusions:**

Our study highlights a shift in SLDs in the United States during the COVID-19 pandemic, showing a decrease in MASLD and increases in MetALD and ALD, with an alarming increase in the prevalence of CSF, likely reflecting lifestyle changes, including physical inactivity and alcohol consumption.

## Graphical abstract

**Figure:**
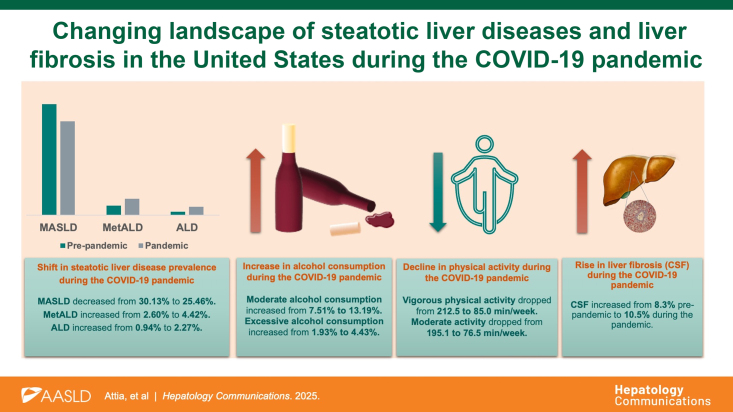

## INTRODUCTION

Recently, a nomenclature shift has occurred in classifying steatotic liver conditions. The term “steatotic liver disease” (SLD) has been introduced to replace “fatty liver disease,” with NAFLD and NASH now referred to as metabolic dysfunction–associated steatotic liver disease (MASLD) and metabolic dysfunction–associated steatohepatitis (MASH), respectively.^1^^,^^2^ With regard to alcohol consumption, MASLD applies to individuals consuming <30 g/d of alcohol for men and <20 g/d for women. A new category, metabolic dysfunction and alcohol-associated liver disease (MetALD), includes moderate alcohol intake, defined as 20–50 g/d for women (140–350 g/wk) and 30–60 g/d for men (210–420 g/wk). This category bridges MASLD and alcohol-associated liver disease (ALD), the latter diagnosed when alcohol consumption exceeds 60 g/d for men and 50 g/d for women, or when individuals without cardiometabolic risk factors (CMRF) consume >30 g/d for men or>20 g/d for women.

Both MASLD and MASH are significant factors contributing to liver-related morbidity and mortality, as well as to the incidence of cancer.^3^^,^^4^ There was a significant rise in the global prevalence of MASLD, increasing from 25.26% (95% CI: 21.59%–29.33%) in the 1990–2006 period to 38.0% (95% CI: 33.71%–42.49%) in the 2016–2019 timeframe. Between 1990 and 2019, the prevalence of MASLD and MASH in North America was estimated to be 31.20% (95% CI: 25.86%–37.08%) and 5.0% (SE: 2.50), respectively.^3^ In addition, ALD poses a significant public health challenge and is a leading cause of liver transplants, resulting in the loss of 11 million life-years globally in 2019.^5^ The rising rates of mortality and disability-adjusted life-years associated with this condition, especially in the United States, are alarming, with economic consequences projected to potentially double by 2040.^5^ Based on a recent meta-analysis,^6^ the pooled prevalence of advanced liver fibrosis, which can lead to liver-related mortality and morbidity, was 3.3% (95% CI: 2.4%-4.2%) worldwide, with a significant increasing trend (*p*=0.004). After Oceania, North America had the highest prevalence at 4.8% (95% CI: 3.8%-5.8%).

After updating terminology and definitions for SLDs, several studies have used the 2017–2020 National Health and Nutrition Examination Survey (NHANES) cycles to assess the prevalence of SLDs, including MASLD, MetALD, and ALD, in US adults. Reported SLD prevalence ranges from ~34.2% to 42.1%, depending on the eligibility criteria applied.^7^^–^^10^

In recent years, several key developments may have influenced the epidemiology of SLDs. The approval and use of new obesity medications could impact obesity rates, subsequently affecting SLD prevalence.^11^ Factors such as physical activity,^12^ stress, depression, lifestyle modifications, and alcohol consumption,^13^^–^^15^ each of which can contribute to the progression of SLD, may have also been altered by the COVID-19 pandemic. Lockdowns and social distancing likely reduced physical activity and negatively impacted mental health, potentially increasing stress and depression. These changes may have contributed to shifts in obesity rates and, consequently, SLDs prevalence during the pandemic.

This study aimed to evaluate the prevalence of SLD with its subcategories and liver stiffness in the United States using NHANES data during the pre-COVID-19 pandemic (2017–2020) and pandemic (2021–2023) periods. The analysis includes overall assessments and subgroup evaluations by demographic and socioeconomic characteristics. In addition, we investigated changes in lifestyle factors—such as body mass index (BMI), physical inactivity, and alcohol consumption—before and during the pandemic as potential contributors to the changing distribution of SLDs.

## METHODS

### Study design and population

This study analyzed NHANES data, a cross-sectional survey assessing the health and nutritional status of the noninstitutionalized civilian population in the United States. NHANES uses a complex, stratified, multistage probability sampling design to obtain a representative sample. Data from 2 cycles were used: the prepandemic cycle (2017–2020) and the pandemic cycle (2021–2023). Participants aged 18 years or older who completed demographic, socioeconomic, clinical, and liver health assessments were included to evaluate trends in liver health over time. Exclusion criteria encompassed cases missing sample weights, as weighting is essential for accurate population-level estimates, and participants without alcohol consumption data or vibration-controlled transient elastography (VCTE) data.

### Data collection and SLD subcategories

NHANES collects data through structured interviews, physical examinations, and laboratory tests in mobile examination centers. This study analyzed key variables across demographic, socioeconomic, behavioral, and liver health domains. Alcohol intake was assessed using the NHANES Alcohol Use Questionnaire (ALQ111, ALQ121, and ALQ130). ALQ111 indicates whether someone has ever had any type of alcoholic drink. Total alcohol consumption in grams per week was calculated by multiplying the drinking frequency from ALQ121 by the average daily drinks from ALQ130. Each drink (12-oz beer, a 5-oz glass of wine, or 1.5 oz liquor) was assumed to contain 14 g of pure alcohol, providing the total weekly intake in grams. The poverty-income ratio was classified as poor (<1), near poor (1–1.9), middle income (2–2.9), and higher income (≥3). Liver health was evaluated using liver stiffness measurement (LSM) and controlled attenuation parameter (CAP) values, both measured through VCTE. VCTE measurements were considered invalid and excluded if they had fewer than 10 complete stiffness measures or an interquartile range/median stiffness ≥30%, per manufacturer guidelines. Liver stiffness (kPa) indicates liver fibrosis, while CAP values (dB/m) assess hepatic steatosis, which served as the primary diagnostic criteria for SLD. Liver disease status was classified into MASLD, MetALD, and ALD, without SLDs (those without steatosis), and other SLDs (non-cardiometabolic conditions like DILI, HCV, etc). After confirming steatosis, alcohol consumption thresholds and CMRFs were used to determine whether participants had MASLD, MetALD, or ALD, as mentioned in the introduction section.^1^^,^^2^

Hepatic steatosis cases were identified using a CAP score cutoff of >285 dB/m^16^ as the primary threshold. Sensitivity analyses were conducted with additional cutoffs of ≥263 dB/m^16^ and ≥248 dB/m^17^ to assess the robustness of the findings. An LSM >8.6 kPa was considered indicative of clinically significant fibrosis (CSF).^16^

CMRFs were defined as BMI ≥25 kg/m^2^, waist circumference ≥94 cm in men or ≥80 cm in women, fasting glucose ≥100 mg/dL, HbA1c ≥5.7%, blood pressure ≥130/85 mm Hg, or HDL cholesterol ≤40 mg/dL in men or ≤50 mg/dL in women. Triglycerides, a standard component of CMRFs, were excluded from the pandemic cycle (2021–2023) selection criteria due to missing data. Therefore, it was not used in both cohorts to ensure comparability. Participants on lipid-lowering medications were considered to have CMRFs.

### Statistical analysis

All statistical analyses accounted for NHANES’ complex survey design, including sample weights, stratification, and clustering, per NHANES guidelines. This ensures that all prevalence estimates and comparisons are representative of the US adult population to reflect national-level impact. Age-adjusted means and prevalences were calculated using the direct method based on the 2000 US Census standard population. Categorical variables (percentages with 95% CI) were compared between prepandemic and pandemic groups using chi-square tests, while continuous variables (means with SE) were compared using independent *t* tests. Univariate and multivariate logistic regression analyses assessed associations between potential risk factors and CSF, with OR and 95% CI calculated for each variable. Statistical significance was set at a 2-tailed *p* value ≤0.05. Analyses were conducted using R (version 4.1.2), with results weighted for generalizability to the US population.

## RESULTS

### Characteristics of participants in NHANES cycles: 2017–2020 (prepandemic) and 2021–2023 (pandemic)

After excluding participants under 18 and those with missing sample weights, 8965 and 6337 individuals from NHANES cycles 2017–2020 and 2021–2023, respectively, were included, yielding a total of 15,302 adults (Table 1). Of these, 1131 (7.4%) did not have valid VCTE data: 2 (0.01%) did not undergo examination, and 1129 (7.4%) had unreliable measurements due to technical or clinical limitations (eg, fasting <3 h, fewer than 10 valid measures obtained, IQR/M>30%, obesity, increased abdominal circumference, or inability to perform a urine pregnancy test), as per NHANES protocol. Thus, 14,171 participants (92.6%) had valid VCTE data. Complete alcohol consumption data were essential for classifying participants into specific SLD subtypes. Consequently, of the 14,171 participants with valid VCTE data, 9399 had complete alcohol data and were assessed for liver disease, with valid VCTE data available for 5557 individuals in the prepandemic cycle (2017–2020) and 3842 in the pandemic cycle (2021–2023). In 2017–2020, 1986 participants had hepatic steatosis (CAP >285 dB); of these, 1973 had at least one CMRF, 57 (0.94%) met ALD criteria, representing 3.1 million (95% CI: 2.1–4.1 million) people in the United States, 1795 (30.13%) were classified as MASLD (99.9 million, 95% CI: 91.6–108.2 million), and 127 (2.60%) as MetALD (8.6 million, 95% CI: 5.7–11.5 million). In 2021–2023, 1275 had hepatic steatosis, with 1263 having at least 1 CMRF; 66 (2.27%) met ALD criteria (7.6 million, 95% CI: 5.4–9.9 million), 1042 (25.46%) were classified as MASLD (85.8 million, 95% CI: 76.2–95.3 million), and 158 (4.42%) as MetALD (14.9 million, 95% CI: 11.7–18.1 million). To further illustrate the population-level impact, we provide population estimates for key demographic, socioeconomic, and health characteristics in Table 2 and Supplemental Table S5, http://links.lww.com/HC9/C116. Baseline characteristics were similar between cycles (Supplemental Figures S1 and S2, http://links.lww.com/HC9/C117, and Table 1). Table 1 presents baseline characteristics for the broader sample (8965 prepandemic and 6337 pandemic participants, before excluding those with missing alcohol data), consistent with NHANES reporting standards. In addition, to assess potential selection bias introduced by exclusions due to missing VCTE data, sample weights, or incomplete alcohol data, we compared baseline characteristics between participants classified for SLDs (n=9399) and those excluded from classification (n=8447) (Supplemental Table S6, http://links.lww.com/HC9/C116). Overall, the 2 groups were largely comparable. While excluded participants had slightly higher BMI (median 28.6 vs. 28.4 kg/m2), greater waist circumference (99.8 vs. 98.5 cm), and a modestly higher obesity prevalence (42.5% vs. 41.6%), these differences were small in magnitude. Clinical measures such as systolic blood pressure (122 vs. 120 mm Hg), levels of physical activity (vigorous activity: median 60 min/wk in both groups), and depressive symptoms were closely matched. Data on determining SLDs and their subcategories based on cutoffs of 263 and 248 are available in Supplemental Figures S3–S6, http://links.lww.com/HC9/C117.

**TABLE 1:** Comparison of baseline characteristics between prepandemic (2017–2020) and pandemic (2021–2023) NHANES participants

Variable	Prepandemic	Pandemic	*p*
Total participants	8965	6337	—
Age at screening (y)	40.96 (0.31)	40.83 (0.50)	0.56
Age categories (%)			
18–34	29.8 (27.89, 31.71)	29.4 (26.85, 31.93)	0.847
35–49	24 (22.38, 25.70)	23.9 (22.02, 25.69)	
50–64	25.6 (24.08, 27.20)	25.1 (23.13, 27.05)	
≥65	20.5 (18.29, 22.74)	21.7 (19.51, 23.82)	
Sex (%)			
Female	50.71 (49.03, 52.39)	50.49 (48.68, 52.31)	0.823
Male	49.29 (47.61, 50.97)	49.51 (47.69, 51.32)	
Race/ethnicity (%)			
Hispanic	18.98 (15.45, 22.50)	19.64 (13.78, 25.51)	0.081
Non-Hispanic Asian	6.41 (4.50, 8.32)	6.88 (4.38, 9.38)	
Non-Hispanic Black	12.14 (9.27, 15.01)	11.58 (9.09, 14.07)	
Non-Hispanic White	58.15 (53.43, 62.86)	55.57 (51.49, 59.65)	
Other	4.33 (3.57, 5.08)	6.32 (5.22, 7.42)	
Weight (kg)	84.99 (0.73)	84.62 (0.83)	0.739
BMI (kg/m^2^)	29.75 (0.22)	29.64 (0.30)	0.744
BMI categories (%)			
Underweight	1.76 (1.24, 2.29)	2.01 (1.50, 2.53)	0.910
Normal weight	25.90 (23.95, 27.86)	26.81 (23.72, 29.91)	
Overweight	30.24 (28.73, 31.75)	30.55 (28.61, 32.48)	
Obesity	42.09 (39.45, 44.73)	40.63 (36.65, 44.61)	
Waist circumference (cm)	99.45 (0.57)	99.20 (0.70)	0.789
Marital status (%)			
Married/living with partner	62.56 (60.18, 64.95)	60.58 (58.58, 62.58)	0.568
Never married	25.00 (22.92, 27.08)	26.71 (24.65, 28.76)	
Widowed/divorced/separated	12.44 (11.22, 13.66)	12.71 (11.26, 14.16)	
Educational status (%)			
High school graduate	25.93 (23.13, 28.73)	25.61 (21.97, 29.25)	0.835
Less than high school graduate	10.39 (8.92, 11.86)	9.29 (7.21, 11.37)	
More than high school graduate	63.68 (60.02, 67.34)	65.11 (60.36, 69.85)	
Poverty-income ratio (%)			
Higher income	39.39 (35.53, 43.24)	36.23 (30.85, 41.61)	0.562
Middle income	27.13 (24.81, 29.46)	28.84 (26.39, 31.29)	
Near poor	18.90 (16.62, 21.19)	19.25 (16.46, 22.05)	
Poor	14.57 (12.68, 16.47)	15.68 (12.64, 18.72)	
Ever smoked 100+ cigarettes (%)			
No	60.29 (57.63, 62.96)	66.83 (62.99, 70.67)	0.013***
Yes	39.71 (37.04, 42.37)	33.17 (29.33, 37.01)	
Currently smoking (%)			
Every day	36.62 (31.85, 41.39)	36.50 (32.33, 40.66)	0.343
Not at all	52.06 (47.23, 56.89)	54.25 (50.57, 57.93)	
Some days	11.32 (9.20, 13.44)	9.26 (7.28, 11.23)	
Physical activity (min/wk)			
Vigorous	212.51 (10.59)	84.99 (8.62)	<0.001***
Moderate	195.06 (6.86)	76.52 (7.95)	<0.001***
Sedentary	387.37 (11.43)	408.64 (7.83)	0.133
Total alcohol consumption (g/wk)	83.84 (11.89)	95.17 (3.54)	0.405
Alcohol consumption levels			
Nondrinkers/light	90.57 (88.93, 92.20)	82.37 (80.48, 84.27)	<0.001
Moderate	7.51 (5.96, 9.05)	13.19 (11.48, 14.91)	
Excessive	1.93 (1.33, 2.52)	4.43 (3.61, 5.25)	
Blood pressure			
Systolic blood pressure (mm Hg)	118.78 (0.32)	118.14 (0.41)	0.206
Diastolic blood pressure (mm Hg)	74.03 (0.27)	74.84 (0.33)	0.046***
Lipid profile			
Total cholesterol (mg/dL)	187.11 (1.26)	189.18 (1.20)	0.283
Direct HDL cholesterol (mg/dL)	53.22 (0.39)	52.85 (0.47)	0.554
Fasting glucose (mg/dL)	106.91 (0.86)	106.66 (1.12)	0.736
HbA1c	5.58 (0.02)	5.61 (0.03)	0.439
HbA1c Categories (%)			
Normal	73.81 (72.45, 75.16)	74.00 (71.22, 76.78)	0.615
Prediabetes	19.50 (18.35, 20.64)	18.78 (16.53, 21.02)	
Diabetes	6.70 (5.83, 7.56)	7.22 (6.21, 8.23)	
Feeling down, depressed, or hopeless (%)			
Nearly every day	2.46 (2.01, 2.90)	3.73 (2.87, 4.59)	<0.001***
More than half the days	4.00 (3.43, 4.58)	5.51 (4.56, 6.45)	
Several days	18.77 (17.24, 20.30)	27.01 (25.54, 28.48)	
Not at all	74.77 (73.46, 76.08)	63.75 (61.89, 65.62)	
Poor appetite or overeating (%)			
Nearly every day	4.30 (3.37, 5.23)	4.95 (3.88, 6.01)	<0.001***
More than half the days	4.72 (3.75, 5.69)	7.04 (6.03, 8.04)	
Several days	16.92 (15.49, 18.35)	22.66 (19.95, 25.36)	
Not at all	74.07 (72.19, 75.94)	65.36 (62.22, 68.49)	

*Notes*: *p* values for categorical variables were calculated using the chi-square test, and for continuous variables using the independent *t* test. Categorical data are presented as percentages with 95% CIs, while continuous data are presented as means with standard error. Significance was considered at *p*<0.05. All data presented are adjusted using NHANES sampling weights for accurate US population representation.

*** *p*-value ≤ 0.05.

Abbreviations: BMI, body mass index; HbA1c, hemoglobin A1c; NHANES, National Health and Nutrition Examination Survey.

**TABLE 2:** Comparison of age-adjusted prevalence of steatotic liver diseases (based on controlled attenuation parameter cutoff score >285) subcategories (percentage) between prepandemic (2017–2020) and pandemic (2021–2023) NHANES participants

	MASLD		MetALD		ALD		No SLDs		Other SLDs		
Variable	Prepandemic	Pandemic	Prepandemic	Pandemic	Prepandemic	Pandemic	Prepandemic	Pandemic	Prepandemic	Pandemic	*p*
Overall	30.13 (27.64, 32.63)	25.46 (22.63, 28.29)	2.60 (1.73, 3.47)	4.42 (3.47, 5.37)	0.94 (0.63, 1.24)	2.27 (1.61, 2.94)	66.27 (63.39, 69.15)	67.43 (65.09, 69.78)	0.06 (0.02, 0.10)	0.42 (0.11, 0.72)	0.003***
Population estimate (CI)	99.9 million (91.6 million–108.2 million)	85.8 million (76.2 million–95.3 million)	8.6 million (5.7 million–11.5 million)	14.9 million (11.7 million–18.1 million)	3.1 million (2.1 million–4.1 million)	7.6 million (5.4 million–9.9 million)	219.8 million (210.1 million–229.3 million)	227.1 million (219.2 million–235.0 million)	0.2 million (0.1 million–0.3 million)	1.4 million (0.4 million–2.4 million)	
Race											
Hispanic	39.18 (35.57, 42.80)	29.65 (22.47, 36.83)	2.13 (1.31, 2.96)	5.07 (2.86, 7.28)	1.57 (0.73, 2.40)	1.76 (0.18, 3.35)	57.04 (53.42, 60.66)	63.13 (56.37, 69.88)	0.08 (0, 0.23)	0.39 (0, 1.00)	0.06
Non-Hispanic Asian	30.44 (25.66, 35.22)	26.29 (14.72, 37.86)	0.77 (0.02, 1.52)	0.80 (0.00, 2.42)	0.33 (0.00, 0.97)	0.00 (0.00, 0.00)	68.46 (63.95, 72.98)	69.41 (60.40, 78.42)	0.00 (0.00, 0.00)	3.50 (0.00, 7.26)	0.465
Non-Hispanic Black	24.25 (21.68, 26.82)	21.35 (14.84, 27.87)	1.87 (1.01, 2.73)	2.77 (1.20, 4.34)	0.40 (0.04, 0.77)	0.66 (0.00, 1.49)	73.25 (70.72, 75.79)	75.22 (69.50, 80.94)	0.22 (0.00, 0.46)	0.00 (0.00, 0.00)	0.468
Non-Hispanic White	28.22 (24.55, 31.88)	24.98 (22.37, 27.58)	2.94 (1.47, 4.40)	4.82 (3.71, 5.94)	0.87 (0.47, 1.28)	2.53 (1.70, 3.37)	67.95 (63.55, 72.35)	67.38 (64.68, 70.07)	0.02 (0.00, 0.07)	0.29 (0.00, 0.66)	0.003***
Other	32.72 (23.49, 41.94)	23.17 (14.75, 31.58)	4.22 (0.00, 8.66)	3.28 (0.52, 6.04)	0.78 (0.00, 1.83)	5.56 (0.17, 10.95)	62.18 (52.56, 71.79)	67.99 (57.18, 78.80)	0.11 (0.00, 0.34)	0.00 (0.00, 0.00)	0.181
Sex											
Female	24.52 (21.62, 27.42)	23.48 (20.84, 26.12)	1.84 (0.73, 2.95)	2.91 (1.58, 4.24)	0.35 (0.03, 0.66)	0.57 (0.11, 1.03)	73.25 (70.30, 76.21)	72.95 (70.01, 75.90)	0.04 (0.00, 0.10)	0.08 (0.00, 0.24)	0.714
Male	35.47 (32.24, 38.69)	27.24 (23.70, 30.77)	3.35 (1.94, 4.75)	5.75 (3.91, 7.60)	1.52 (0.93, 2.11)	3.85 (2.71, 5.00)	59.59 (55.81, 63.38)	62.44 (59.49, 65.39)	0.07 (0.00, 0.15)	0.71 (0.17, 1.25)	0.002***
Socioeconomic status											
Higher income	26.07 (21.81, 30.34)	23.39 (18.76, 28.01)	2.87 (1.21, 4.53)	4.75 (2.88, 6.61)	0.51 (0.05, 0.98)	1.37 (0.61, 2.13)	70.50 (65.73, 75.27)	70.07 (65.91, 74.24)	0.04 (0.00, 0.12)	0.42 (0.00, 0.90)	0.284
Middle income	34.82 (29.99, 39.64)	29.24 (25.18, 33.30)	2.83 (0.96, 4.71)	4.13 (2.37, 5.89)	0.99 (0.18, 1.80)	2.68 (1.20, 4.17)	61.28 (56.35, 66.21)	63.17 (58.43, 67.92)	0.08 (0.00, 0.21)	0.77 (0.00, 1.69)	0.125
Near poor	31.60 (28.09, 35.11)	28.48 (23.83, 33.13)	2.12 (1.25, 2.98)	3.33 (1.50, 5.16)	1.74 (0.93, 2.55)	2.77 (1.20, 4.34)	64.42 (60.62, 68.22)	65.42 (59.68, 71.15)	0.13 (0.00, 0.30)	0.00 (0.00, 0.00)	0.27
Poor	28.74 (23.49, 33.98)	24.47 (19.96, 28.97)	1.93 (0.86, 3.00)	5.48 (3.32, 7.64)	1.56 (0.65, 2.47)	2.76 (0.92, 4.60)	67.71 (62.15, 73.26)	67.29 (62.34, 72.25)	0.07 (0.00, 0.20)	0.00 (0.00, 0.00)	0.076
Age categories											
18–34	22.02 (18.38, 25.66)	17.71 (13.20, 22.21)	1.26 (0.67, 1.85)	2.86 (1.62, 4.10)	0.70 (0.16, 1.24)	2.03 (0.61, 3.44)	75.91 (71.95, 79.87)	77.14 (72.55, 81.74)	0.11 (0.01, 0.21)	0.26 (0.00, 0.61)	0.033***
35–49	33.01 (29.32, 36.71)	27.90 (24.76, 31.03)	3.46 (1.51, 5.40)	5.36 (3.37, 7.35)	1.01 (0.42, 1.61)	1.95 (0.83, 3.08)	62.50 (58.18, 66.82)	63.98 (59.51, 68.45)	0.02 (0.00, 0.06)	0.81 (0.07, 1.55)	0.023***
50–64	38.15 (33.43, 42.87)	33.58 (28.54, 38.63)	3.35 (1.31, 5.39)	5.37 (3.65, 7.08)	1.18 (0.62, 1.74)	3.13 (1.99, 4.27)	57.28 (52.46, 62.11)	57.85 (52.72, 62.99)	0.04 (0.00, 0.10)	0.07 (0.00, 0.19)	0.066

*Notes*: *p* values were calculated using the chi-square test. Data are presented as percentages with 95% CIs. Significance was considered at *p*<0.05. All data presented are adjusted using NHANES sampling weights for accurate US population representation.

Abbreviations: ALD, alcohol- associated liver disease; MASLD, metabolic dysfunction–associated steatotic liver disease; MetALD, metabolic dysfunction and alcohol-associated liver disease; NHANES, National Health and Nutrition Examination Survey; SLD, steatotic liver disease.

### Temporal changes in physical activity and alcohol consumption during the COVID-19 pandemic

Vigorous physical activity decreased from 212.51 min/wk prepandemic to 84.99 during the pandemic (*p*<0.001), and moderate physical activity dropped from 195.06 to 76.52 min (*p*<0.001) (Table 1). While there was no significant change in the age-adjusted prevalence of current smokers between the cycles, a significant reduction in the prevalence of smoking 100+ cigarettes in a lifetime was observed, dropping from 39.71% prepandemic to 33.17% pandemic (*p*=0.013). Total alcohol consumption increased nonsignificantly from 83.84 to 95.17 g/wk from prepandemic to pandemic periods (*p*=0.405). However, when categorized by intake level, nondrinkers/light drinkers (<140 g/wk for females, <210 g/wk for males) decreased, while moderate (140–350 g/wk for females, 210–420 g/wk for males) and excessive drinkers (>350 g/wk for females, >420 g/wk for males) increased significantly during the pandemic (*p*<0.001). The percentage of individuals reporting appetite-related changes increased significantly during the pandemic (*p*<0.001), with more individuals experiencing either poor appetite or overeating. Despite lifestyle changes, there were no significant differences in BMI, weight, waist circumference, total cholesterol, HDL cholesterol, fasting glucose, HbA1c, or blood pressure between the 2 periods.

### Temporal changes in the prevalence of SLDs during the COVID-19 pandemic

#### Overall trend

Age-adjusted prevalence of SLD subcategories changed significantly during the pandemic compared with the prepandemic cycle, across all 3 CAP cutoffs (248, 263, and 285) evaluated (*p*=0.003, 0.004, and 0.003, respectively) (Table 2 and Supplemental Tables S1 and S2, http://links.lww.com/HC9/C116). Across all cutoffs, MASLD decreased, while MetALD and ALD increased. Notably, at a CAP cutoff of 285, the proportion of individuals diagnosed with ALD rose to 2.27% (95% CI: 1.61%–2.94%) during the pandemic, compared with 0.94% (95% CI: 0.63%–1.24%) prepandemic. MASLD prevalence was lower in the pandemic at 25.46% (95% CI: 22.63%–28.29%) versus 30.13% (95% CI: 27.64%–32.63%) prepandemic.

#### Age and gender subgroup analysis

Significant changes in SLD subcategories were observed in the 18–34 (*p*=0.033) and 35–49 (*p*=0.023) age groups and in males (*p*=0.002), but not in the 50–64 age group (*p*=0.066) or in females (*p*=0.714) (Table 2). In males, ALD prevalence more than doubled from 1.52% prepandemic to 3.85% during the pandemic, while MetALD rose from 3.35% to 5.75%. Conversely, MASLD declined from 35.47% to 27.24%. The decrease in MASLD prevalence was greater in the 35–49 group (5.11% vs. 4.31%) than in the 18–34 group, whereas ALD prevalence increased more in the 18–34 group (1.33% vs. 0.94%) than in the 35–49 group. Sensitivity analysis at CAP cutoffs of 248 and 263 showed consistent trends, with significant changes observed only in the 50–64 age group, in both genders at 248, and only in males at 263 (Supplemental Tables S1 and S2, http://links.lww.com/HC9/C116).

#### Race/ethnicity and socioeconomic subgroup analysis

SLD subcategories showed statistically significant changes between the 2 cycles only in the non-Hispanic Whites (*p*=0.003). For the 263 cutoff, non-Hispanic Whites and non-Hispanic Blacks showed significant changes, but for the 248 cutoff, none of the racial/ethnic groups had significant changes. We observed a numerical decrease in MASLD prevalence and an increase in MetALD and ALD prevalence across socioeconomic groups, although statistical significance was not reached for any of the income categories, except for the “Near Poor” group at a 263 cutoff (Table 2 and Supplemental Tables S1 and S2, http://links.lww.com/HC9/C116).

### Temporal changes in hepatic steatosis and fibrosis during the COVID-19 pandemic

LSM increased during the pandemic from 5.82 to 6.10 kPa with borderline statistical significance (*p*=0.149). The increase in LSM was significant among those categorized as poor income (from 5.80 to 6.82 kPa, *p*=0.04) and in individuals aged 50–64 (from 6.19 to 7.00 kPa, *p*=0.019) (Supplemental Figure S7, http://links.lww.com/HC9/C117, and Supplemental Table S3, http://links.lww.com/HC9/C116).

**FIGURE 1:**
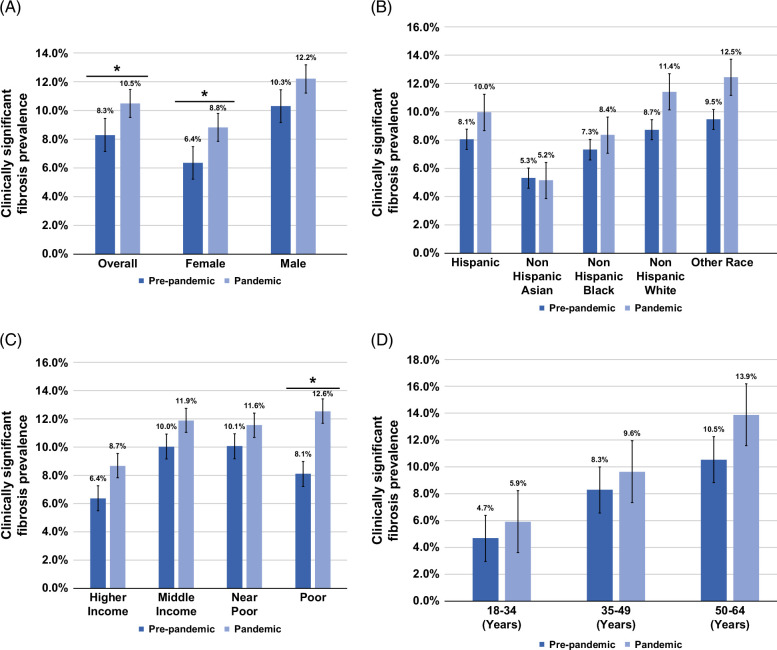
Comparison of age-adjusted clinically significant fibrosis prevalence among US adults in prepandemic (2017–2020) versus pandemic (2021–2023). (A) Shows overall and sex-specific comparisons (female and male); (B) compares racial/ethnic groups (Hispanic, Non-Hispanic Asian, Non-Hispanic Black, Non-Hispanic White, and Other); (C) examines socioeconomic groups (higher income, middle income, near poor, and poor); and (D) highlights age groups (18–34, 35–49, and 50–64). Estimates are shown with 95% CIs, and significant prepandemic versus pandemic differences (*p*≤0.05) are marked with an asterisk (*).

**FIGURE 2:**
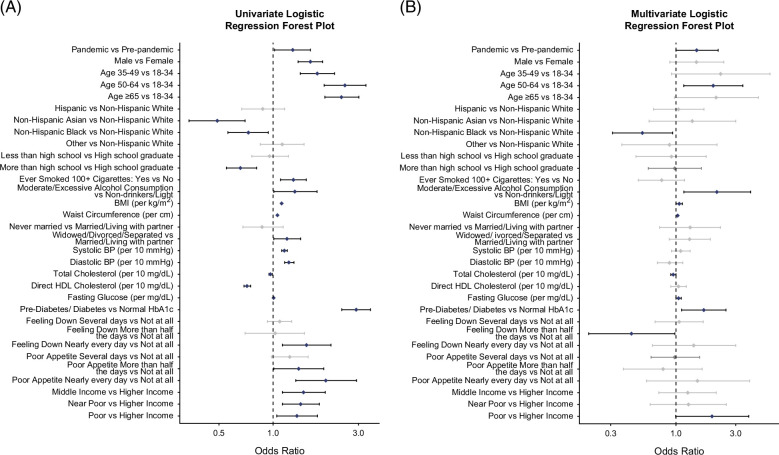
Predictors of clinically significant fibrosis: Forest plots show ORs from univariate (A) and multivariate (B) logistic regression. Blue markers denote significant predictors (*p*≤0.05), and gray markers indicate nonsignificant predictors.

The prevalence of individuals with CSF increased from 8.3% to 10.5% (*p*=0.028) (Figure 1 and Supplemental Table S3, http://links.lww.com/HC9/C116). Further stratified analysis by hepatic steatosis thresholds (CAP>285, 263, and 248 dB/m) showed significant increases in CSF prevalence among individuals with ALD (eg, CAP >285: from 15.2% to 33.0%, *p*=0.01), whereas there were numerically higher but nonsignificant increases of CSF in MASLD (16.3%–19.9%, *p*=0.07) and MetALD (12.6%–18.4%, *p*=0.4). Detailed results are available in Supplemental Table S3, http://links.lww.com/HC9/C116. The multivariable logistic regression model showed that the COVID-19 pandemic (vs. prepandemic) is independently associated with CSF (adjusted odds ratio [aOR]: 1.47, 95% CI: 1.00, 2.17) after adjusting for relevant confounders (Figure 2 and Supplemental Table S4, http://links.lww.com/HC9/C116). Notably, moderate/excessive (vs. mild-no) alcohol intake (aOR: 2.13, 95% CI: 1.15, 3.95) was associated with CSF. In addition, older age (aOR: 1.98, 95% CI: 1.15, 3.41 for 50–64 y vs. 18–34 y), higher BMI (aOR: 1.06, 95% CI: 1.001, 1.12), larger waist circumference (aOR: 1.03, 95% CI: 1.001, 1.05), and prediabetes/diabetes (aOR: 1.67, 95% CI: 1.11, 2.51) were each independently associated with CSF.

**FIGURE 3:**
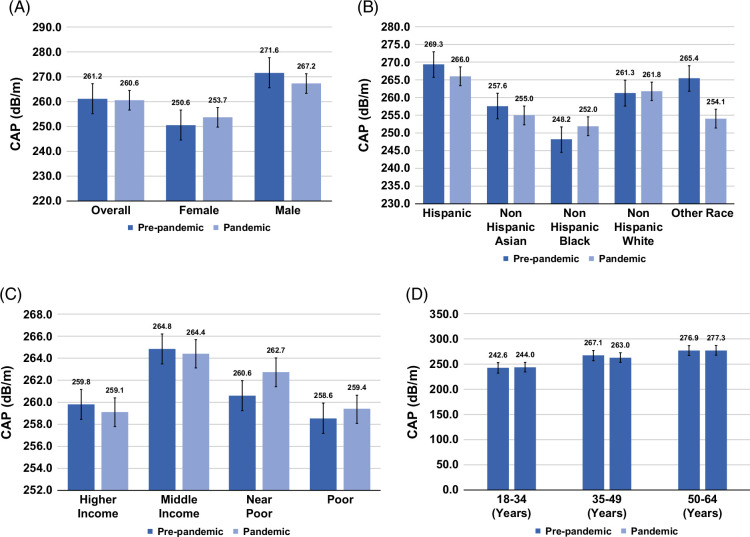
Comparison of age-adjusted mean controlled attenuation parameter scores among US adults in prepandemic (2017–2020) versus pandemic (2021–2023). (A) Shows overall and sex-specific comparisons (female and male); (B) compares racial/ethnic groups (Hispanic, Non-Hispanic Asian, Non-Hispanic Black, Non-Hispanic White, and Other); (C) examines socioeconomic groups (higher income, middle income, near poor, and poor); and (D) highlights age groups (18–34, 35–49, and 50–64). Estimates are presented with error bars representing the SE.

CAP scores remained stable with no significant change (260.56 dB/m during the pandemic vs. 261.17 dB/m prepandemic, *p*=0.804), including across all subgroups (gender, race/ethnicity, and SES) (Figure 3 and Supplemental Table S3, http://links.lww.com/HC9/C116).

## DISCUSSION

Our study provides valuable insights into the changing landscape of SLDs in the United States during the COVID-19 pandemic. We found a 4.6% decline in MASLD prevalence among US adults, from 30.1% prepandemic (2017–2020) to 25.5% during the pandemic (2021–2023). Conversely, MetALD prevalence rose from 2.6% to 4.4%, and ALD from 0.9% to 2.3%. We observed a nonsignificant increase in LSM, but the prevalence of those with CSF increased by 2.2% during the pandemic.

When evaluating potential factors influencing the burden of SLDs, no differences were found in anthropometric measures like BMI and waist circumference between the prepandemic and pandemic periods. Recent years have seen a rise in the use of glucagon-like peptide-1 (GLP-1) receptor agonists and dual GLP-1 and gastric inhibitory polypeptide receptor agonists,^18^ which may have implications for obesity management and potentially influence the prevalence of SLD. Estimates suggest that around 1 in 8 adults in the United States have reported using GLP-1 agonists at some point,^19^ indicating a significant uptake. However, the long-term impact of these agents on obesity rates and associated outcomes remains to be fully understood.^20^ In line with a recent report utilizing data from the 2021–2023 NHANES cycle,^21^ which observed a nonsignificant downward trend in obesity compared with the 2017–2020 cycle, our findings similarly showed a slight numerical increase in the percentage of individuals with a normal BMI (from 25.9% to 26.8%) and a decrease in those categorized as obese (from 42.09% to 40.63%). While this could be an important factor for the observed decline in MASLD prevalence and the rise in individuals classified as having no SLDs in our results, it should be interpreted with caution, as BMI changes were not statistically significant.

This trend of the decline in MASLD and the increase in individuals with no SLDs contrasts with the rise in MetALD and ALD during the same periods. Various aspects of life may have been affected during the pandemic, including physical inactivity, alcohol consumption, and the prevalence of stress, anxiety, and depression.^22^^–^^25^ The pandemic likely caused a reduction in physical activity due to restrictions and temporary closures.^23^ In our results, there was a statistically significant decrease in both vigorous and moderate physical activity per week between these 2 periods. In addition, reports suggest that alcohol consumption may have risen,^24^^,^^25^ potentially affecting liver health and metabolic conditions. Our results also showed an increased percentage of both moderate (1.76 times) and excessive (2.3 times) drinkers during the pandemic. Moreover, heightened levels of stress, anxiety, and depression during the pandemic^22^ may have influenced not only mental health but also lifestyle choices and overall physical well-being. Prior research underscores that healthy diets, sufficient physical activity, and higher education are linked to lower MASLD risk, reinforcing the need for lifestyle interventions.^26^ Overall, our data also show that feelings of depression and hopelessness became more common during the pandemic, with a noticeable increase in the frequency of these emotions compared with the prepandemic period.

Liver fibrosis is the most potent predictor of liver disease morbidity and mortality, with liver disease mortality risk 4-fold higher among those with CSF.^27^ There was a 2.2% increase in the prevalence of CSF in the US general population during the pandemic, which was further confirmed in our multivariate logistic regression analysis, and the pandemic period was associated with a modest but notable increase in the odds (OR: 1.47) for CSF. The analysis also highlighted that moderate or excessive alcohol consumption (OR: 2.13) was a significant predictor of CSF, suggesting that increased use of moderate or excessive alcohol consumption during the COVID-19 pandemic, in part, contributed to the increased prevalence of CSF in the US general population. Furthermore, PNPLA3 rs738409 interacts with modifiable factors (eg, alcohol, diet) to influence liver mortality. Though genetic data were unavailable here, our findings—increased alcohol use and CSF—suggest environmental exposures exacerbated inherited risks.^28^

These results highlight the complex interplay of demographic factors, such as race/ethnicity and SES, along with lifestyle factors affecting liver health before and during the pandemic. This year, resmetirom became the first FDA-approved drug for noncirrhotic MASH with moderate to advanced liver fibrosis.^29^^,^^30^ There are other drugs under evaluation in clinical trials for the treatment of MASH with fibrosis, such as survodutide^31^ and tirzepatide.^32^ A recent phase III clinical trial^33^ demonstrated that semaglutide treatment in patients with MASH and moderate or advanced liver fibrosis significantly improved liver histology by achieving steatohepatitis resolution without fibrosis worsening, reducing fibrosis without steatohepatitis worsening, and attaining a combined outcome of both steatohepatitis resolution and fibrosis reduction. With the approval and availability of these drugs, as well as wider use of GLP-1 or dual GLP-1 and gastric inhibitory polypeptide receptor agonists, the landscape of SLDs may further evolve. Future research should also continue to monitor lifestyle factors influenced by the COVID-19 pandemic, such as alcohol consumption, and assess SLD subcategories over time in the postpandemic period.

The study has several limitations. This study lacks histological confirmation of SLDs through liver biopsy, but we relied on CAP from VCTE to evaluate steatosis. LSM is a noninvasive test to measure liver fibrosis, but results can overestimate the degree of liver fibrosis after acute alcohol use, which may reflect acute inflammation instead of liver fibrosis. In addition, VCTE measurement failures, particularly in participants with higher BMI, may introduce bias due to technical challenges, potentially excluding those with severe hepatic steatosis or fibrosis. This may result in conservative estimates of SLD prevalence in high-BMI populations. Alcohol consumption was self-reported over the past year, introducing bias from misreporting or recall errors, which may affect estimates for ALD and MetALD. Metabolic risk factors relied on self-reported diagnoses or single-point lab results with possible ascertainment bias. In addition, triglycerides—a standard CMRF—were excluded in the pandemic cycle (2021–2023) due to missing data, leading to their omission from both cycles for comparability.

Another potential limitation of this project is related to the impact of the pandemic on NHANES cycle design and sampling methods.^34^ A new 2-year sample design was implemented for the pandemic cycle to reduce interviewer-participant contact. Unlike previous cycles, there was no oversampling by race, Hispanic origin, or income, and person-level oversampling by age groups was changed. While the overall sample size remained similar to previous cycles, subgroup sizes changed. For example, the proportion of non-Hispanic Whites was higher, while the proportion of non-Hispanic Blacks was lower. In addition, a considerable proportion of participants were excluded due to missing critical data (sample weights, VCTE measurements, or alcohol data). Although NHANES’s sampling design is robust, this could introduce potential selection bias, skewing results toward healthier or more compliant individuals. Nonetheless, excluded participants were overall comparable to those included (Supplemental Table S6, http://links.lww.com/HC9/C116), suggesting limited risk of major selection bias and supporting the generalizability of our findings to the broader US adult population.

In conclusion, our study sheds light on the shifting prevalence of SLDs and CSF in the United States during the COVID-19 pandemic, highlighting key changes in the prevalence of SLD, its subcategories, and CSF. Our findings show a significant reduction in MASLD prevalence and a corresponding increase in MetALD and ALD, which reflects higher rates of moderate and excessive alcohol use during the pandemic. In addition, the observed increase in CSF appears driven by multiple independent factors, including the pandemic period, alcohol use, BMI, waist circumference, prediabetes/diabetes, and socioeconomic status, emphasizing the multifactorial nature of liver disease progression. These findings underscore the complex interplay of demographic, metabolic, genetic factors, and lifestyle factors affecting liver health and highlight the need for public health interventions that address lifestyle factors contributing to the progression of liver disease to prevent rising liver disease mortality.

## Supplementary Material
