# Supplementary material for: Changing landscape of steatotic liver diseases and liver fibrosis in the United States during the COVID-19 pandemic
Source: Hepatol Commun. 2025 Sep 5;9(9):e0806. doi: 10.1097/HC9.0000000000000806 (PMC12412732; doi:10.1097/HC9.0000000000000806)
Supplement: Supplementary file 1 [file hc9-9-e0806-s001.docx]

**Supplementary Table 1. Comparison of Age-Adjusted Prevalence of Steatotic Liver Diseases (Based on Controlled Attenuation Parameter Cutoff Score ≥263) subcategories (Percentage) between pre-pandemic (2017–2020) and pandemic (2021–2023) NHANES participants.**

| Variable | MASLD | | MetALD | | ALD | | No SLDs | | Other SLDs | | P-value |
| --- | --- | --- | --- | --- | --- | --- | --- | --- | --- | --- | --- |
|  | Pre-pandemic | Pandemic | Pre-pandemic | Pandemic | Pre-pandemic | Pandemic | Pre-pandemic | Pandemic | Pre-pandemic | Pandemic |  |
| Overall | 40.63 (38.07, 43.20) | 35.80 (32.70, 38.89) | 3.95 (3.14, 4.75) | 5.90 (4.72, 7.09) | 1.39 (0.95, 1.83) | 2.74 (2.02, 3.45) | 53.72 (50.88, 56.57) | 55.01 (52.25, 57.77) | 0.31 (0.08, 0.54) | 0.55 (0.22, 0.88) | 0.004*** |
| Race/Ethnicity |  |  |  |  |  |  |  |  |  |  |  |
| Hispanic | 51.48 (47.90, 55.06) | 43.57 (36.62, 50.51) | 2.53 (1.57, 3.49) | 5.79 (3.56, 8.01) | 1.88 (1.01, 2.75) | 2.26 (0.65, 3.87) | 43.77 (39.81, 47.74) | 47.68 (41.18, 54.17) | 0.34 (0.09, 0.58) | 0.71 (-0.08, 1.50) | 0.095 |
| Non-Hispanic Asian | 41.02 (37.18, 44.86) | 40.18 (26.71, 53.66) | 1.41 (0.05, 2.77) | 5.87 (2.53, 9.21) | 0.33 (0, 0.97) | 0.00 (0.00, 0.00) | 56.24 (52.00, 60.47) | 50.45 (38.34, 62.56) | 1.01 (0, 2.26) | 3.50 (0.00, 7.26) | 0.21 |
| Non-Hispanic Black | 36.31 (34.14, 38.48) | 31.20 (24.50, 37.90) | 2.70 (1.79, 3.62) | 3.86 (2.13, 5.59) | 0.57 (0.13, 1.01) | 1.89 (0.20, 3.59) | 59.85 (57.53, 62.17) | 63.05 (57.64, 68.46) | 0.57 (0.25, 0.89) | 0.00 (0.00, 0.00) | 0.01*** |
| Non-Hispanic White | 37.75 (34.05, 41.45) | 34.45 (31.46, 37.45) | 4.62 (3.13, 6.12) | 6.40 (5.00, 7.79) | 1.42 (0.71, 2.13) | 2.87 (2.01, 3.74) | 55.98 (51.73, 60.24) | 55.88 (52.80, 58.96) | 0.22 (0.00, 0.52) | 0.40 (0.00, 0.80) | 0.024*** |
| Other | 45.54 (37.52, 53.57) | 30.95 (22.95, 38.94) | 6.11 (0.71, 11.51) | 3.94 (1.76, 6.12) | 1.84 (0.28, 3.41) | 5.75 (0.58, 10.93) | 46.39 (38.48, 54.30) | 59.03 (49.61, 68.45) | 0.11 (0.00, 0.34) | 0.33 (0.00, 0.80) | 0.105 |
| Gender |  |  |  |  |  |  |  |  |  |  |  |
| Female | 35.84 (32.46, 39.23) | 32.30 (29.31, 35.28) | 2.98 (1.84, 4.11) | 4.31 (3.09, 5.53) | 0.47 (0.13, 0.80) | 0.95 (0.45, 1.46) | 60.51 (56.99, 64.02) | 62.15 (59.36, 64.94) | 0.21 (0.06, 0.36) | 0.29 (0.01, 0.57) | 0.096 |
| Male | 45.17 (41.95, 48.39) | 38.95 (35.32, 42.59) | 4.91 (3.40, 6.42) | 7.30 (5.17, 9.43) | 2.29 (1.37, 3.21) | 4.40 (3.12, 5.68) | 47.23 (43.75, 50.72) | 48.56 (45.25, 51.88) | 0.39 (0.08, 0.71) | 0.79 (0.26, 1.31) | 0.012*** |
| Age Categories |  |  |  |  |  |  |  |  |  |  |  |
| 18-34 | 29.54 (25.87, 33.21) | 26.66 (20.81, 32.51) | 1.85 (0.99, 2.72) | 4.20 (2.85, 5.55) | 1.04 (0.41, 1.66) | 2.49 (1.15, 3.83) | 67.02 (62.79, 71.24) | 66.34 (60.65, 72.03) | 0.55 (0.08, 1.02) | 0.31 (0.00, 0.68) | 0.053 |
| 35-49 | 45.64 (41.31, 49.96) | 38.81 (34.47, 43.16) | 5.01 (2.87, 7.15) | 6.77 (4.36, 9.18) | 1.76 (0.86, 2.67) | 2.23 (1.12, 3.34) | 47.39 (42.94, 51.85) | 51.30 (46.19, 56.42) | 0.19 (0.01, 0.38) | 0.88 (0.13, 1.64) | 0.085 |
| 50-64 | 49.97 (45.86, 54.07) | 45.15 (42.26, 48.05) | 5.52 (3.50, 7.53) | 7.20 (5.37, 9.03) | 1.36 (0.78, 1.93) | 3.88 (2.38, 5.37) | 43.05 (39.10, 46.99) | 43.35 (40.13, 46.56) | 0.11 (0.00, 0.22) | 0.43 (0.00, 0.89) | 0.005*** |
| Socioeconomic Status |  |  |  |  |  |  |  |  |  |  |  |
| Higher Income | 36.28 (31.31, 41.25) | 32.93 (28.03, 37.82) | 3.90 (2.05, 5.74) | 6.46 (4.22, 8.71) | 0.63 (0.13, 1.13) | 1.75 (1.01, 2.50) | 58.74 (53.23, 64.26) | 58.36 (53.85, 62.87) | 0.45 (0.00, 1.14) | 0.50 (0.00, 1.00) | 0.090 |
| Middle Income | 43.61 (38.75, 48.48) | 39.83 (35.02, 44.64) | 4.22 (2.37, 6.06) | 5.78 (3.57, 7.99) | 1.62 (0.46, 2.77) | 2.98 (1.45, 4.50) | 50.37 (45.75, 54.99) | 50.25 (45.14, 55.36) | 0.19 (0.00, 0.38) | 1.17 (0.14, 2.19) | 0.163 |
| Near Poor | 44.98 (40.77, 49.20) | 35.98 (31.23, 40.73) | 3.09 (1.63, 4.55) | 5.19 (3.30, 7.08) | 2.98 (1.24, 4.72) | 3.26 (1.61, 4.92) | 48.68 (44.67, 52.70) | 55.57 (50.09, 61.05) | 0.27 (0.00, 0.59) | 0.00 (0.00, 0.00) | 0.015*** |
| Poor | 39.74 (35.89, 43.59) | 37.76 (29.90, 45.61) | 4.54 (3.01, 6.06) | 5.97 (3.74, 8.20) | 2.17 (1.24, 3.10) | 3.91 (1.69, 6.12) | 53.27 (49.27, 57.28) | 52.30 (45.76, 58.84) | 0.28 (0.01, 0.54) | 0.07 (0.00, 0.20) | 0.200 |

Abbreviations MASLD, metabolic-associated steatotic liver disease; MetALD, metabolic alcohol-related liver disease; ALD, alcohol-related liver disease; SLD, steatotic liver disease.

Notes: P-values were calculated using the Chi-square test. Data are presented as Percentage with 95 confidence intervals. Significance was considered at P < 0.05. All data presented are adjusted using NHANES sampling weights for accurate U.S. population representation.

**Supplementary Table 2. Comparison of Age-Adjusted Prevalence of Steatotic Liver Diseases (Based on Controlled Attenuation Parameter Cutoff Score ≥248) subcategories (Percentage) between pre-pandemic (2017–2020) and pandemic (2021–2023) NHANES participants.**

| Variable | MASLD | | MetALD | | ALD | | No SLDs | | Other SLDs | | P-value |
| --- | --- | --- | --- | --- | --- | --- | --- | --- | --- | --- | --- |
|  | Pre-pandemic | Pandemic | Pre-pandemic | Pandemic | Pre-pandemic | Pandemic | Pre-pandemic | Pandemic | Pre-pandemic | Pandemic |  |
| Overall | 46.80 (44.31, 49.30) | 42.16 (38.71, 45.61) | 4.64 (3.39, 5.89) | 6.78 (5.59, 7.96) | 1.64 (1.10, 2.19) | 3.14 (2.46, 3.83) | 46.11 (43.28, 48.95) | 46.83 (43.61, 50.05) | 0.80 (0.30, 1.30) | 1.09 (0.66, 1.53) | 0.003*** |
| Race/Ethnicity |  |  |  |  |  |  |  |  |  |  |  |
| Hispanic | 57.99 (54.87, 61.10) | 49.73 (43.37, 56.09) | 2.70 (1.77, 3.62) | 6.14 (3.88, 8.39) | 2.08 (1.10, 3.05) | 2.97 (1.17, 4.78) | 36.33 (32.94, 39.73) | 39.58 (33.82, 45.35) | 0.91 (0.31, 1.50) | 1.57 (0.42, 2.72) | 0.066 |
| Non-Hispanic Asian | 47.81 (43.77, 51.85) | 47.72 (35.62, 59.81) | 1.41 (0.05, 2.77) | 5.87 (2.53, 9.21) | 0.33 (0.00, 0.97) | 0.33 (0.00, 1.00) | 48.67 (43.87, 53.47) | 41.06 (31.52, 50.59) | 1.78 (0.00, 3.69) | 5.02 (1.19, 8.86) | 0.200 |
| Non-Hispanic Black | 43.34 (41.04, 45.63) | 40.58 (31.56, 49.60) | 3.20 (2.22, 4.17) | 4.13 (2.25, 6.01) | 0.68 (0.22, 1.14) | 2.30 (0.42, 4.18) | 51.77 (49.21, 54.33) | 52.69 (45.49, 59.89) | 1.01 (0.48, 1.55) | 0.30 (0.00, 0.95) | 0.066 |
| Non-Hispanic White | 43.65 (39.98, 47.32) | 40.11 (36.10, 44.11) | 5.61 (3.40, 7.81) | 7.67 (6.30, 9.04) | 1.69 (0.92, 2.46) | 3.09 (2.15, 4.04) | 48.35 (44.10, 52.60) | 48.54 (44.27, 52.82) | 0.71 (0.00, 1.47) | 0.59 (0.14, 1.04) | 0.058 |
| Other | 52.27 (45.03, 59.51) | 39.41 (30.51, 48.30) | 6.11 (0.71, 11.51) | 4.25 (1.81, 6.69) | 2.74 (0.53, 4.96) | 6.67 (0.51, 12.84) | 38.64 (31.89, 45.39) | 47.28 (37.85, 56.70) | 0.23 (0.00, 0.51) | 2.39 (0.00, 5.01) | 0.081 |
| Gender |  |  |  |  |  |  |  |  |  |  |  |
| Female | 42.38 (39.10, 45.65) | 39.12 (35.51, 42.73) | 3.77 (2.34, 5.19) | 4.94 (3.69, 6.19) | 0.55 (0.18, 0.93) | 1.29 (0.77, 1.81) | 52.80 (49.08, 56.53) | 53.61 (49.95, 57.28) | 0.50 (0.23, 0.78) | 1.04 (0.27, 1.81) | 0.027*** |
| Male | 50.96 (47.69, 54.23) | 44.90 (41.21, 48.60) | 5.49 (3.75, 7.24) | 8.39 (6.59, 10.19) | 2.70 (1.63, 3.78) | 4.85 (3.71, 6.00) | 39.78 (36.38, 43.17) | 40.72 (37.42, 44.03) | 1.07 (0.28, 1.86) | 1.13 (0.54, 1.73) | 0.011*** |
| Age Categories |  |  |  |  |  |  |  |  |  |  |  |
| 18-34 | 34.70 (31.30, 38.11) | 33.74 (27.46, 40.03) | 2.31 (1.13, 3.50) | 5.27 (3.96, 6.57) | 1.46 (0.57, 2.35) | 2.79 (1.27, 4.31) | 59.92 (55.60, 64.24) | 56.73 (50.33, 63.14) | 1.61 (0.52, 2.70) | 1.47 (0.37, 2.56) | 0.105 |
| 35-49 | 51.28 (47.30, 55.27) | 43.80 (39.14, 48.47) | 5.95 (2.81, 9.08) | 7.24 (4.74, 9.73) | 1.86 (0.88, 2.85) | 2.82 (1.91, 3.74) | 40.55 (36.33, 44.77) | 45.15 (40.19, 50.12) | 0.36 (0.06, 0.66) | 0.98 (0.19, 1.77) | 0.139 |
| 50-64 | 58.46 (53.44, 63.48) | 52.51 (48.83, 56.20) | 6.21 (4.13, 8.29) | 8.38 (6.17, 10.60) | 1.59 (0.91, 2.28) | 4.15 (2.66, 5.65) | 33.50 (28.89, 38.11) | 34.27 (30.84, 37.69) | 0.24 (0.07, 0.40) | 0.68 (0.10, 1.27) | 0.012*** |
| Socioeconomic Status |  |  |  |  |  |  |  |  |  |  |  |
| Higher Income | 42.48 (37.71, 47.24) | 39.71 (34.58, 44.85) | 5.32 (2.32, 8.31) | 7.78 (5.13, 10.43) | 0.97 (0.10, 1.83) | 1.94 (1.01, 2.87) | 50.78 (45.45, 56.12) | 49.37 (44.17, 54.57) | 0.45 (0.00, 1.14) | 1.20 (0.65, 1.74) | 0.096 |
| Middle Income | 51.03 (45.65, 56.40) | 44.08 (39.42, 48.73) | 4.28 (2.37, 6.19) | 6.35 (4.09, 8.61) | 1.86 (0.67, 3.06) | 3.52 (2.11, 4.93) | 41.89 (36.99, 46.78) | 44.48 (39.50, 49.46) | 0.94 (0.13, 1.76) | 1.57 (0.46, 2.69) | 0.114 |
| Near Poor | 49.35 (44.85, 53.84) | 45.58 (40.19, 50.96) | 3.40 (1.93, 4.86) | 5.70 (3.82, 7.58) | 3.30 (1.51, 5.10) | 3.26 (1.61, 4.92) | 43.26 (39.06, 47.46) | 44.46 (38.42, 50.50) | 0.69 (0.18, 1.20) | 1.00 (0.00, 2.34) | 0.267 |
| Poor | 44.72 (40.06, 49.38) | 42.75 (34.36, 51.13) | 4.84 (3.27, 6.41) | 6.55 (4.35, 8.74) | 2.29 (1.39, 3.18) | 6.10 (1.82, 10.38) | 47.69 (42.73, 52.65) | 44.54 (38.23, 50.85) | 0.46 (0.15, 0.77) | 0.07 (0.00, 0.20) | 0.114 |

Abbreviations MASLD, metabolic-associated steatotic liver disease; MetALD, metabolic alcohol-related liver disease; ALD, alcohol-related liver disease; SLD, steatotic liver disease.

Notes: P-values were calculated using the Chi-square test. Data are presented as Percentage with 95 confidence intervals. Significance was considered at P < 0.05. All data presented are adjusted using NHANES sampling weights for accurate U.S. population representation.

**Supplementary Table 3. Comparison of Age-Adjusted Mean Liver Stiffness and Controlled Attenuation Parameter Between Pre-Pandemic (2017-2020) and Pandemic (2021-2023) NHANES Participants.**

| Variables | Pre-Pandemic | Pandemic | P Value |
| --- | --- | --- | --- |
| Liver Stiffness, kPa, overall | 5.82 (0.11) | 6.10 (0.17) | 0.149 |
| Clinically significant fibrosis (LSM>8.6) |  |  |  |
| No | 91.7 (90.21, 93.19) | 89.5 (88.45, 90.52) | 0.028*** |
| Yes | 8.3 (6.81, 9.79) | 10.5 (9.48, 11.55) |  |
| Clinically significant fibrosis stratified by CAP > 285 |  |  |  |
| MASLD | 16.34 (15.05, 17.63) | 19.92 (19.83, 20.01) | 0.07 |
| MetALD | 12.64 (3.76, 21.51) | 18.40 (16.47, 20.33) | 0.4 |
| ALD | 15.22 (3.85, 26.59) | 33.03 (27.77, 38.29) | 0.01*** |
| Clinically significant fibrosis stratified by CAP ≥ 263 |  |  |  |
| MASLD | 13.13 (11.32, 14.94) | 16.23 (14.54, 17.91) | 0.06 |
| MetALD | 10.88 (2.96, 18.80) | 15.5 (12.37, 18.66) | 0.22 |
| ALD | 12.50 (0.88, 24.11) | 26.84 (20.55, 33.13) | 0.03*** |
| Clinically significant fibrosis stratified by CAP ≥ 248 |  |  |  |
| MASLD | 11.74 (10.32, 13.16) | 14.36 (13.31, 15.42) | 0.07 |
| MetALD | 10.24 (3.49, 17.00) | 13.40 (11.26, 15.54) | 0.31 |
| ALD | 11.27 (0.37, 22.17) | 25.40 (21.09, 29.79) | 0.03*** |
| Liver Stiffness, kPa, Gender |  |  |  |
| Male | 6.27 (0.15) | 6.54 (0.19) | 0.288 |
| Female | 5.35 (0.13) | 5.64 (0.17) | 0.161 |
| Age Categories |  |  |  |
| 18-34 | 5.52 (0.18) | 5.24 (0.09) | 0.168 |
| 35-49 | 5.77 (0.17) | 6.19 (0.25) | 0.174 |
| 50-64 | 6.19 (0.13) | 7.00 (0.30) | 0.019*** |
| Liver Stiffness, kPa, Race/Ethnicity |  |  |  |
| Hispanic | 5.66 (0.12) | 6.04 (0.29) | 0.244 |
| Non-Hispanic Asian | 5.16 (0.13) | 4.84 (0.16) | 0.16 |
| Non-Hispanic Black | 5.79 (0.14) | 5.83 (0.17) | 0.945 |
| Non-Hispanic White | 5.89 (0.16) | 6.30 (0.20) | 0.11 |
| Other | 6.44 (0.42) | 6.35 (0.40) | 0.906 |
| Liver Stiffness, kPa, SES |  |  |  |
| Higher Income | 5.57 (0.15) | 5.77 (0.15) | 0.35 |
| Middle Income | 6.04 (0.20) | 6.39 (0.29) | 0.32 |
| Near Poor | 6.05 (0.26) | 5.86 (0.14) | 0.536 |
| Poor | 5.80 (0.12) | 6.82 (0.49) | 0.04*** |
| CAP, dB/m, overall | 261.17 (1.64) | 260.56 (1.82) | 0.804 |
| CAP, dB/m, Gender |  |  |  |
| Male | 271.57 (2.11) | 267.20 (2.10) | 0.115 |
| Female | 250.61 (1.69) | 253.66 (2.06) | 0.177 |
| Age Categories |  |  |  |
| 18-34 | 242.60 (2.64) | 243.95 (2.76) | 0.726 |
| 35-49 | 267.06 (2.41) | 263.03 (2.32) | 0.236 |
| 50-64 | 276.88 (2.45) | 277.34 (2.18) | 0.89 |
| CAP, dB/m, Race/Ethnicity |  |  |  |
| Hispanic | 269.33 (2.08) | 265.98 (3.55) | 0.355 |
| Non-Hispanic Asian | 257.61 (1.86) | 254.98 (6.24) | 0.841 |
| Non-Hispanic Black | 248.20 (1.74) | 251.95 (3.12) | 0.37 |
| Non-Hispanic White | 261.29 (2.77) | 261.79 (2.47) | 0.918 |
| Other | 265.41 (4.58) | 254.12 (3.86) | 0.097 |
| CAP, dB/m, kPa, SES |  |  |  |
| Higher Income | 259.81 (3.34) | 259.10 (3.01) | 0.817 |
| Middle Income | 264.82 (2.48) | 264.40 (2.22) | 0.899 |
| Near Poor | 260.59 (2.20) | 262.72 (2.95) | 0.555 |
| Poor | 258.55 (3.02) | 259.36 (4.66) | 0.997 |

Abbreviations: CAP, controlled attenuation parameter; LSM, liver stiffness measurement; SES, socioeconomic status.

**Notes:** Data are presented as mean (standard error) or Percentage with 95 confidence intervals. P-values were calculated using the Independent T-test or the Chi-square test. Significance was considered at P < 0.05. All data presented are adjusted using NHANES sampling weights for accurate U.S. population representation.

**Supplementary Table 4. Predictors of Clinically Significant Fibrosis: Univariate and Multivariate Logistic Regression Analysis**

|  | Univariate Logistic regression | | Multivariate Logistic regression | |
| --- | --- | --- | --- | --- |
| Variables | **OR (95 CI)** | **P-value** | **OR (95 CI)** | **P-value** |
| Cycle |  |  |  |  |
| Pre-pandemic | Reference |  | Reference | 0.050*** |
| Pandemic | 1.29 (1.02, 1.62) | 0.031*** | 1.47 (1.00, 2.17) |  |
| Gender |  |  |  |  |
| Female | Reference |  | Reference | 0.120 |
| Male | 1.62 (1.38, 1.89) | <0.001*** | 1.46 (0.89, 2.40) |  |
| Age (years) |  |  |  |  |
| 18-34 | Reference |  | Reference |  |
| 35-49 | 1.77 (1.416, 2.21) | <0.001*** | 2.28 (0.92, 5.64) | 0.069 |
| 50-64 | 2.52 (1.927, 3.30) | <0.001*** | 1.98 (1.15, 3.41) | 0.019*** |
| ≥65 | 2.42 (1.95, 3.01) | <0.001*** | 2.10 (0.97, 4.56) | 0.057 |
| Race/Ethnicity |  |  |  |  |
| Non-Hispanic White | Reference |  | Reference |  |
| Hispanic | 0.88 (0.67, 1.16) | 0.375 | 1.05 (0.66, 1.67) | 0.800 |
| Non-Hispanic Asian | 0.49 (0.34, 0.70) | <0.001*** | 1.35 (0.61, 3.00) | 0.400 |
| Non-Hispanic Black | 0.73 (0.56, 0.94) | 0.017*** | 0.54 (0.31, 0.94) | 0.034*** |
| Other | 1.13 (0.85, 1.49) | 0.365 | 0.89 (0.37, 2.13) | 0.800 |
| Educational status |  |  |  |  |
| High school graduate | Reference |  | Reference |  |
| Less than high school graduate | 0.96 (0.756, 1.22) | 0.747 | 0.92 (0.48, 1.74) | 0.800 |
| More than high school graduate | 0.66 (0.548, 0.81) | <0.001*** | 0.98 (0.60, 1.59) | >0.900 |
| Ever Smoked 100+ Cigarettes |  |  |  |  |
| No | Reference |  | Reference | 0.200 |
| Yes | 1.30 (1.10, 1.53) | 0.002*** | 0.77 (0.50, 1.17) |  |
| Physical Activity (minutes/week) |  |  |  |  |
| Vigorous | 1 (0.99, 1.00) | 0.839 | - | - |
| Moderate | 0.99 (0.995, 1.00) | 0.560 | - | - |
| Sedentary | 1.00 (1, 1.00) | 0.161 | - | - |
| Alcohol Consumption Levels |  |  |  |  |
| Nondrinkers/Light | Reference |  | Reference |  |
| Moderate/Excessive | 1.33 (1.01, 1.76) | 0.041 | 2.13 (1.15, 3.95) | 0.022*** |
| BMI (kg/m2) | 1.12 (1.11, 1.13) | <0.001*** | 1.06 (1.00, 1.12) | 0.039*** |
| Waist Circumference (cm) | 1.06 (1.05, 1.06) | <0.001*** | 1.03 (1.00, 1.05) | 0.034*** |
| Marital status |  |  |  |  |
| Married/Living with partner | Reference |  | Reference |  |
| Never married | 0.87 (0.676, 1.138) | 0.315 | 1.30 (0.74, 2.26) | 0.300 |
| Widowed/Divorced/Separated | 1.20 (1.007, 1.433) | 0.042*** | 1.29 (0.88, 1.88) | 0.200 |
| Blood Pressure |  |  |  |  |
| Systolic Blood Pressure (mmHg/10) | 1.16 (1.12, 1.20) | 0.004*** | 1.09 (0.92, 1.30) | 0.300 |
| Diastolic Blood Pressure (mmHg/10) | 1.23 (1.16, 1.31) | <0.001*** | 0.89 (0.71, 1.13) | 0.300 |
| Lipid Profile |  |  |  |  |
| Total Cholesterol (per 10 mg/dL) | 0.97 (0.95, 0.99) | 0.004*** | 0.95 (0.91, 1.00) | 0.046*** |
| Direct HDL Cholesterol (per 10 mg/dL) | 0.72 (0.69, 0.75) | <0.001*** | 1.05 (0.91, 1.21) | 0.5 |
| Fasting Glucose (mg/dL) | 1.01 (1.01, 1.01) | <0.001*** | 1.05 (1.00, 1.10) | 0.041*** |
| HbA1c Categories |  |  |  |  |
| Normal | Reference |  | Reference | 0.021*** |
| Pre-Diabetes/Diabetes | 2.91 (2.42, 3.50) | <0.001*** | 1.67 (1.11, 2.51) |  |
| Feeling Down Depressed or Hopeless | |  |  |  |
| Not at all | Reference |  | Reference |  |
| Several days | 1.09 (0.93, 1.27) | 0.3 | 1.06 (0.68, 1.65) | 0.8 |
| More than half the days | 1.03 (0.70, 1.50) | 0.9 | 0.44 (0.20, 0.98) | 0.045*** |
| Nearly every day | 1.54 (1.13, 2.10) | 0.008*** | 1.39 (0.65, 3.00) | 0.3 |
| Poor Appetite or Overeating |  |  |  |  |
| Not at all | Reference |  | Reference |  |
| Several days | 1.24 (0.98, 1.57) | 0.064 | 0.98 (0.63, 1.54) | >0.9 |
| More than half the days | 1.39 (1.01, 1.92) | 0.041*** | 0.79 (0.38, 1.61) | 0.5 |
| Nearly every day | 1.98 (1.34, 2.93) | <0.001*** | 1.49 (0.58, 3.85) | 0.4 |
| Poverty-Income Ratio |  |  |  |  |
| Higher Income | Reference |  | Reference |  |
| Middle Income | 1.48 (1.13, 1.96) | 0.006*** | 1.24 (0.73, 2.10) | 0.4 |
| Near Poor | 1.43 (1.13, 1.81) | 0.004*** | 1.26 (0.62, 2.53) | 0.5 |
| Poor | 1.36 (1.05, 1.77) | 0.022*** | 1.94 (0.99, 3.81) | 0.05*** |

Abbreviations: BMI, body mass index; HDL, high-density lipoprotein; HBA1C, hemoglobin A1C; OR, Odds Ratio; CI, Confidence Interval

**Notes:** Significance was considered at P < 0.05. All data presented are adjusted using NHANES sampling weights for accurate U.S. population representation.

**Supplementary Table 5. Population Estimates and Comparison of Age-Adjusted Prevalence of Steatotic Liver Diseases (Based on Controlled Attenuation Parameter Cutoff Score >285) Subcategories (Percentage) Between Pre-Pandemic (2017–2020) and Pandemic (2021–2023) NHANES Participants.**

| **Variable** | **MASLD** | | **MetALD** | | | **ALD** | | **No SLDs** | | **Other SLDs** | | **P-value** |
| --- | --- | --- | --- | --- | --- | --- | --- | --- | --- | --- | --- | --- |
|  | Pre-pandemic | Pandemic | Pre-pandemic | | Pandemic | Pre-pandemic | Pandemic | Pre-pandemic | Pandemic | Pre-pandemic | Pandemic |  |
| **Overall** | 30.13 (27.64, 32.63) | 25.46 (22.63, 28.29) | 2.60 (1.73, 3.47) | | 4.42 (3.47, 5.37) | 0.94 (0.63, 1.24) | 2.27 (1.61, 2.94) | 66.27 (63.39, 69.15) | 67.43 (65.09, 69.78) | 0.06 (0.02, 0.10) | 0.42 (0.11, 0.72) | 0.003*** |
| **Population Estimate (CI)** | 99.9 M (91.6–108.2 M) | 85.8 M (76.2–95.3 M) | 8.6 M (5.7–11.5 M) | | 14.9 M (11.7–18.1 M) | 3.1 M (2.1–4.1 M) | 7.6 M (5.4–9.9 M) | 219.8 M (210.1–229.3 M) | 227.1 M (219.2–235.0 M) | 0.2 M (0.1–0.3 M) | 1.4 M (0.4–2.4 M) |  |
| **Race** |  |  |  |  | |  |  |  |  |  |  |  |
| Hispanic | 39.18 (35.57, 42.80) | 29.65 (22.47, 36.83) | 2.13 (1.31, 2.96) | | 5.07 (2.86, 7.28) | 1.57 (0.73, 2.40) | 1.76 (0.18, 3.35) | 57.04 (53.42, 60.66) | 63.13 (56.37, 69.88) | 0.08 (0, 0.23) | 0.39 (0, 1.00) | 0.06 |
|  | 24.64 M (22.37–26.92 M) | 19.6 M (14.85–24.34 M) | 1.34 M (0.82–1.86 M) | | 3.35 M (1.89–4.81 M) | 0.99 M (0.46–1.51 M) | 1.16 M (0.12–2.21 M) | 35.88 M (33.6–38.16 M) | 41.73 M (37.26–46.19 M) | 0.05 M (0.0–0.14 M) | 0.26 M (0.0–0.66 M) |  |
| Non-Hispanic Asian | 30.44 (25.66, 35.22) | 26.29 (14.72, 37.86) | 0.77 (0.02, 1.52) | | 0.80 (0.00, 2.42) | 0.33 (0.00, 0.97) | 0.00 (0.00, 0.00) | 68.46 (63.95, 72.98) | 69.41 (60.40, 78.42) | 0.00 (0.00, 0.00) | 3.50 (0.00, 7.26) | 0.465 |
|  | 6.48 M (5.47–7.5 M) | 6.1 M (3.42–8.78 M) | 6.1 M (3.42–8.78 M) | | 0.19 M (0.0–0.56 M) | 0.07 M (0.0–0.21 M) | 0.0 M (0.0–0.0 M) | 14.58 M (13.62–15.54 M) | 16.1 M (14.01–18.19 M) | 0.0 M (0.0–0.0 M) | 0.81 M (0.0–1.68 M) |  |
| Non-Hispanic Black | 24.25 (21.68, 26.82) | 21.35 (14.84, 27.87) | 1.87 (1.01, 2.73) | | 2.77 (1.20, 4.34) | 0.40 (0.04, 0.77) | 0.66 (0.00, 1.49) | 73.25 (70.72, 75.79) | 75.22 (69.50, 80.94) | 0.22 (0.00, 0.46) | 0.00 (0.00, 0.00) | 0.468 |
|  | 9.77 M (8.74–10.81 M) | 8.33 M (5.79–10.87 M) | 0.75 M (0.41–1.1 M) | | 1.08 M (0.47–1.69 M) | 0.16 M (0.02–0.31 M) | 0.26 M (0.0–0.58 M) | 29.52 M (28.5–30.54 M) | 29.34 M (27.11–31.57 M) | 0.09 M (0.0–0.19 M) | 0.0 M (0.0–0.0 M) |  |
| Non-Hispanic White | 28.22 (24.55, 31.88) | 24.98 (22.37, 27.58) | 2.94 (1.47, 4.40) | | 4.82 (3.71, 5.94) | 0.87 (0.47, 1.28) | 2.53 (1.70, 3.37) | 67.95 (63.55, 72.35) | 67.38 (64.68, 70.07) | 0.02 (0.00, 0.07) | 0.29 (0.00, 0.66) | 0.003*** |
|  | 54.41 M (47.33–61.46 M) | 46.76 M (41.88–51.63 M) | 5.67 M (2.83–8.48 M) | | 9.02 M (6.95–11.12 M) | 1.68 M (0.91–2.47 M) | 4.74 M (3.18–6.31 M) | 131.01 M (122.52–139.49 M) | 126.14 M (121.08–131.17 M) | 0.04 M (0.0–0.13 M) | 0.54 M (0.0–1.24 M) |  |
| Other | 32.72 (23.49, 41.94) | 23.17 (14.75, 31.58) | 4.22 (0.00, 8.66) | | 3.28 (0.52, 6.04) | 0.78 (0.00, 1.83) | 5.56 (0.17, 10.95) | 62.18 (52.56, 71.79) | 67.99 (57.18, 78.80) | 0.11 (0.00, 0.34) | 0.00 (0.00, 0.00) | 0.181 |
|  | 4.71 M (3.38–6.04 M) | 4.94 M (3.14–6.73 M) | 0.61 M (0.0–1.25 M) | | 0.7 M (0.11–1.29 M) | 0.11 M (0.0–0.26 M) | 1.18 M (0.04–2.33 M) | 8.95 M (7.57–10.34 M) | 14.48 M (12.18–16.78 M) | 0.02 M (0.0–0.05 M) | 0.0 M (0.0–0.0 M) |  |
| **Gender** |  |  |  |  | |  |  |  |  |  |  |  |
| Female | 24.52 (21.62, 27.42) | 23.48 (20.84, 26.12) | 1.84 (0.73, 2.95) | | 2.91 (1.58, 4.24) | 0.35 (0.03, 0.66) | 0.57 (0.11, 1.03) | 73.25 (70.30, 76.21) | 72.95 (70.01, 75.90) | 0.04 (0.00, 0.10) | 0.08 (0.00, 0.24) | 0.714 |
|  | 41.22 M (36.34–46.09 M) | 39.92 M (35.43–44.4 M) | 3.09 M (1.23–4.96 M) | | 4.95 M (2.69–7.21 M) | 0.59 M (0.05–1.11 M) | 0.97 M (0.19–1.75 M) | 123.13 M (118.17–128.11 M) | 124.02 M (119.02–129.03 M) | 0.07 M (0.0–0.17 M) | 0.14 M (0.0–0.41 M) |  |
| Male | 35.47 (32.24, 38.69) | 27.24 (23.70, 30.77) | 3.35 (1.94, 4.75) | | 5.75 (3.91, 7.60) | 1.52 (0.93, 2.11) | 3.85 (2.71, 5.00) | 59.59 (55.81, 63.38) | 62.44 (59.49, 65.39) | 0.07 (0.00, 0.15) | 0.71 (0.17, 1.25) | 0.002*** |
|  | 57.99 M (52.71–63.26 M) | 45.44 M (39.53–51.32 M) | 5.48 M (3.17–7.77 M) | | 9.59 M (6.52–12.68 M) | 2.49 M (1.52–3.45 M) | 6.42 M (4.52–8.34 M) | 97.43 M (91.25–103.63 M) | 104.15 M (99.23–109.07 M) | 0.11 M (0.0–0.25 M) | 1.18 M (0.28–2.08 M) |  |
| **Socioeconomic Status** |  |  |  | |  |  |  |  |  |  |  |  |
| Higher Income | 26.07 (21.81, 30.34) | 23.39 (18.76, 28.01) | 2.87 (1.21, 4.53) | | 4.75 (2.88, 6.61) | 0.51 (0.05, 0.98) | 1.37 (0.61, 2.13) | 70.50 (65.73, 75.27) | 70.07 (65.91, 74.24) | 0.04 (0.00, 0.12) | 0.42 (0.00, 0.90) | 0.284 |
|  | 34.05 M (28.48–39.62 M) | 28.54 M (22.89–34.17 M) | 3.75 M (1.58–5.92 M) | | 5.79 M (3.51–8.06 M) | 0.67 M (0.07–1.28 M) | 1.67 M (0.74–2.6 M) | 92.07 M (85.84–98.3 M) | 85.49 M (80.41–90.57 M) | 0.05 M (0.0–0.16 M) | 0.51 M (0.0–1.1 M) |  |
| Middle Income | 34.82 (29.99, 39.64) | 29.24 (25.18, 33.30) | 2.83 (0.96, 4.71) | | 4.13 (2.37, 5.89) | 0.99 (0.18, 1.80) | 2.68 (1.20, 4.17) | 61.28 (56.35, 66.21) | 63.17 (58.43, 67.92) | 0.08 (0.00, 0.21) | 0.77 (0.00, 1.69) | 0.125 |
|  | 31.34 M (26.99–35.68 M) | 28.39 M (24.45–32.33 M) | 2.55 M (0.86–4.24 M) | | 4.01 M (2.3–5.72 M) | 0.89 M (0.16–1.62 M) | 2.6 M (1.17–4.05 M) | 55.15 M (50.72–59.59 M) | 61.34 M (56.74–65.95 M) | 0.07 M (0.0–0.19 M) | 0.75 M (0.0–1.64 M) |  |
| Near Poor | 31.60 (28.09, 35.11) | 28.48 (23.83, 33.13) | 2.12 (1.25, 2.98) | | 3.33 (1.50, 5.16) | 1.74 (0.93, 2.55) | 2.77 (1.20, 4.34) | 64.42 (60.62, 68.22) | 65.42 (59.68, 71.15) | 0.13 (0.00, 0.30) | 0.00 (0.00, 0.00) | 0.27 |
|  | 19.81 M (17.61–22.01 M) | 18.46 M (15.44–21.47 M) | 1.33 M (0.78–1.87 M) | | 2.16 M (0.97–3.34 M) | 1.09 M (0.58–1.6 M) | 1.79 M (0.78–2.81 M) | 40.39 M (38.01–42.77 M) | 42.39 M (38.67–46.11 M) | 0.08 M (0.0–0.19 M) | 0.0 M (0.0–0.0 M) |  |
| Poor | 28.74 (23.49, 33.98) | 24.47 (19.96, 28.97) | 1.93 (0.86, 3.00) | | 5.48 (3.32, 7.64) | 1.56 (0.65, 2.47) | 2.76 (0.92, 4.60) | 67.71 (62.15, 73.26) | 67.29 (62.34, 72.25) | 0.07 (0.00, 0.20) | 0.00 (0.00, 0.00) | 0.076 |
|  | 13.88 M (11.35–16.41 M) | 12.92 M (10.54–15.3 M) | 0.93 M (0.42–1.45 M) | | 2.89 M (1.75–4.03 M) | 0.75 M (0.31–1.19 M) | 1.46 M (0.49–2.43 M) | 32.7 M (30.02–35.38 M) | 35.53 M (32.92–38.15 M) | 0.03 M (0.0–0.1 M) | 0.0 M (0.0–0.0 M) |  |
| **Age Categories** |  |  |  | |  |  |  |  |  |  |  |  |
| 18-34 | 22.02 (18.38, 25.66) | 17.71 (13.20, 22.21) | 1.26 (0.67, 1.85) | | 2.86 (1.62, 4.10) | 0.70 (0.16, 1.24) | 2.03 (0.61, 3.44) | 75.91 (71.95, 79.87) | 77.14 (72.55, 81.74) | 0.11 (0.01, 0.21) | 0.26 (0.00, 0.61) | 0.033*** |
|  | 21.76 M (18.16–25.35 M) | 17.53 M (13.07–21.99 M) | 1.24 M (0.66–1.83 M) | | 2.83 M (1.6–4.06 M) | 0.69 M (0.16–1.23 M) | 2.01 M (0.6–3.41 M) | 75.0 M (71.09–78.91 M) | 76.37 M (71.82–80.92 M) | 0.11 M (0.01–0.21 M) | 0.26 M (0.0–0.6 M) |  |
| 35-49 | 33.01 (29.32, 36.71) | 27.90 (24.76, 31.03) | 3.46 (1.51, 5.40) | | 5.36 (3.37, 7.35) | 1.01 (0.42, 1.61) | 1.95 (0.83, 3.08) | 62.50 (58.18, 66.82) | 63.98 (59.51, 68.45) | 0.02 (0.00, 0.06) | 0.81 (0.07, 1.55) | 0.023*** |
|  | 26.28 M (23.34–29.22 M) | 22.46 M (19.93–24.98 M) | 2.75 M (1.2–4.3 M) | | 4.31 M (2.71–5.92 M) | 0.8 M (0.33–1.28 M) | 1.57 M (0.67–2.48 M) | 49.75 M (46.31–53.19 M) | 51.5 M (47.91–55.1 M) | 0.02 M (0.0–0.05 M) | 0.65 M (0.06–1.25 M) |  |
| 50-64 | 38.15 (33.43, 42.87) | 33.58 (28.54, 38.63) | 3.35 (1.31, 5.39) | | 5.37 (3.65, 7.08) | 1.18 (0.62, 1.74) | 3.13 (1.99, 4.27) | 57.28 (52.46, 62.11) | 57.85 (52.72, 62.99) | 0.04 (0.00, 0.10) | 0.07 (0.00, 0.19) | 0.066 |
|  | 32.35 M (28.35–36.35 M) | 28.38 M (24.12–32.64 M) | 2.84 M (1.11–4.57 M) | | 4.54 M (3.08–5.98 M) | 1.0 M (0.53–1.48 M) | 2.64 M (1.68–3.61 M) | 48.57 M (44.49–52.67 M) | 48.88 M (44.55–53.23 M) | 0.03 M (0.0–0.08 M) | 0.06 M (0.0–0.16 M) |  |

Abbreviations MASLD, metabolic-associated steatotic liver disease; MetALD, metabolic alcohol- associated liver disease; ALD, alcohol- associated liver disease; SLD, steatotic liver disease; M, million.

**Notes:** P-values were calculated using the Chi-square test. Data are presented as Percentage with 95 confidence intervals. Significance was considered at P < 0.05. All data presented are adjusted using NHANES sampling weights for accurate U.S. population representation.

**Supplementary Table 6. Comparison of Baseline Characteristics Between U.S. Adults Included in Steatotic Liver Disease Classification (n = 9,399) and Those Excluded (n = 8,447) Due to Missing VCTE data, Sampling Weights or Alcohol Data (NHANES 2017–2023)”.**

|  | level | Excluded Participants (n=8447) | Included Participants (n=9399) |
| --- | --- | --- | --- |
| Cycle (%) | Pandemic | 4311 (51.0) | 3842 (40.9) |
|  | Pre-pandemic | 4136 (49.0) | 5557 (59.1) |
| Gender (%) | Female | 4767 (56.4) | 4704 (50.0) |
|  | Male | 3680 (43.6) | 4695 (50.0) |
| Age (median [IQR]) |  | 57.00 [37.00, 70.00] | 49.00 [33.00, 63.00] |
| Age Group (%) | 18-34 | 1835 (21.7) | 2622 (27.9) |
|  | 35-49 | 1533 (18.1) | 2186 (23.3) |
|  | 50-64 | 2120 (25.1) | 2553 (27.2) |
|  | ≥65 | 2959 (35.0) | 2038 (21.7) |
| Race/Ethnicity (%) | Hispanic | 1637 (19.4) | 1902 (20.2) |
|  | Non-Hispanic Asian | 949 (11.2) | 666 (7.1) |
|  | Non-Hispanic Black | 1725 (20.4) | 1875 (19.9) |
|  | Non-Hispanic White | 3654 (43.3) | 4420 (47.0) |
|  | Other Race | 482 (5.7) | 536 (5.7) |
| Educational status (%) | High school graduate | 2016 (25.3) | 1958 (21.7) |
|  | Less than high school graduate | 1755 (22.0) | 1044 (11.5) |
|  | More than high school graduate | 4189 (52.6) | 6038 (66.8) |
| Marital status (%) | Married/Living with partner | 4262 (53.5) | 5153 (57.0) |
|  | Never married | 1454 (18.3) | 1966 (21.8) |
|  | Widowed/Divorced/Separated | 2250 (28.2) | 1920 (21.2) |
| BMI (kg/m2) (median [IQR]) |  | 28.60 [24.60, 33.90] | 28.40 [24.60, 33.40] |
| BMI Group (%) | Underweight | 112 (2.0) | 146 (1.6) |
|  | Normal weight | 1387 (24.4) | 2358 (25.2) |
|  | Overweight | 1767 (31.1) | 2952 (31.6) |
|  | Obesity | 2410 (42.5) | 3893 (41.6) |
| Waist Circumference (cm) |  | 99.80 [88.60, 111.90] | 98.50 [87.70, 110.50] |
| Ever Smoked 100+ Cigarettes (%) | No | 5229 (62.1) | 5448 (58.0) |
|  | Yes | 3188 (37.9) | 3944 (42.0) |
| Currently Smoking (%) | Not at all | 2011 (63.1) | 2247 (57.0) |
|  | Some days | 222 (7.0) | 394 (10.0) |
|  | Every day | 955 (30.0) | 1303 (33.0) |
| Physical Activity (minutes/week) |  |  |  |
| Vigorous (median [IQR]) |  | 60.00 [30.00, 120.00] | 60.00 [30.00, 120.00] |
| Moderate (median [IQR]) |  | 60.00 [30.00, 120.00] | 60.00 [30.00, 180.00] |
| Sedentary (median [IQR]) |  | 300.00 [180.00, 480.00] | 300.00 [180.00, 480.00] |
| Total alcohol grams week (median [IQR]) |  | 8.75 [2.66, 65.62]* | 17.50 [2.69, 84.00] |
| Blood Pressure |  |  |  |
| Systolic Blood Pressure (median [IQR]) |  | 122.00 [110.00, 137.00] | 120.00 [110.00, 132.00] |
| Diastolic Blood Pressure (median [IQR]) |  | 72.00 [65.00, 80.00] | 74.00 [67.00, 81.25] |
| Lipid Profile |  |  |  |
| Total Cholesterol (mg/dL) (median [IQR]) |  | 179.00 [152.00, 208.00] | 184.00 [159.00, 212.00] |
| Direct HDL Cholesterol (mg/dL) (median [IQR]) |  | 50.00 [42.00, 60.00] | 52.00 [43.00, 63.00] |
| Fasting Glucose (mg/dL) (median [IQR]) |  | 103.00 [95.00, 117.00] | 101.00 [95.00, 110.00] |
| HBA1C (median [IQR]) |  | 5.60 [5.30, 6.10] | 5.50 [5.20, 5.80] |
| HbA1c Categories (%) | Pre-Diabetes | 1732 (31.8) | 2340 (26.0) |
|  | Normal | 2732 (50.1) | 5769 (64.0) |
|  | Diabetes | 989 (18.1) | 904 (10.0) |
| Feeling Down Depressed or Hopeless (%) | More than half the days | 234 (5.3) | 448 (4.8) |
|  | Nearly every day | 185 (4.2) | 298 (3.2) |
|  | Not at all | 3207 (72.5) | 6654 (70.9) |
|  | Several days | 799 (18.1) | 1982 (21.1) |
| Poor Appetite or Overeating (%) | More than half the days | 270 (6.1) | 565 (6.0) |
|  | Nearly every day | 256 (5.8) | 412 (4.4) |
|  | Not at all | 3187 (72.0) | 6688 (71.3) |
|  | Several days | 712 (16.1) | 1719 (18.3) |
| Poverty-Income Ratio (median [IQR]) |  | 2.01 [1.09, 3.80] | 2.80 [1.42, 5.00] |
| Poverty-Income Ratio (%) | Higher Income | 1551 (23.2) | 2940 (35.5) |
|  | Middle Income | 1814 (27.1) | 2327 (28.1) |
|  | Near Poor | 1847 (27.6) | 1730 (20.9) |
|  | Poor | 1478 (22.1) | 1292 (15.6) |

Abbreviations: BMI, body mass index; HDL, high-density lipoprotein; HBA1C, hemoglobin A1C.

* Among excluded participants, 5,388 had missing alcohol consumption data.

**Supplementary Figures:**

**Supplementary Figure 1.** Flowchart of Participant Selection and Classification for Steatotic Liver Disease (CAP > 285 dB) in NHANES 2021-2023.

**Supplementary Figure 2.** Flowchart of Participant Selection and Classification for Steatotic Liver Disease (CAP > 285 dB) in NHANES 2017-2020.

**Supplementary Figure 3.** Flowchart of Participant Selection and Classification for Steatotic Liver Disease (CAP ≥263 dB) in NHANES 2021-2023.

**Supplementary Figure 4.** Flowchart of Participant Selection and Classification for Steatotic Liver Disease (CAP ≥263 dB) in NHANES 2017-2020.

**Supplementary Figure 5.** Flowchart of Participant Selection and Classification for Steatotic Liver Disease (CAP > 248 dB) in NHANES 2021-2023.

**Supplementary Figure 6.** Flowchart of Participant Selection and Classification for Steatotic Liver Disease (CAP ≥ 248 dB) in NHANES 2017-2020.

**Supplementary Figure 7.** Comparison of Age-Adjusted Mean Liver Stiffness Measurement (kPa) Among U.S. Population aged 18 Years or Older and Across Demographic Subgroups in Pre-Pandemic (2017–2020) and Pandemic (2021–2023) NHANES Cycles. **Panel A** displays the overall and gender-specific comparison (Female and Male); **Panel B** shows the racial/ethnic group comparison (Hispanic, Non-Hispanic Asian, Non-Hispanic Black, Non-Hispanic White, and Other); **Panel C** illustrates the socioeconomic group comparison (Higher Income, Middle Income, Near Poor, and Poor); and **Panel D** highlights the age group comparison (18-34, 35-49, and 50-64). Estimates are presented with error bars representing the standard error. Statistically significant differences between pre-pandemic and pandemic periods are indicated by an asterisk (*) with a p-value of ≤ 0.05.
