# Supplementary material for: Changing landscape of steatotic liver diseases and liver fibrosis in the United States during the COVID-19 pandemic
Source: Hepatol Commun. 2025 Sep 5;9(9):e0806. doi: 10.1097/HC9.0000000000000806 (PMC12412732; doi:10.1097/HC9.0000000000000806)

Supplementary Figures:

Supplementary Figure 1. Flowchart of Participant Selection and Classification for Steatotic Liver Disease (CAP > 285 dB) in NHANES 2021-2023.

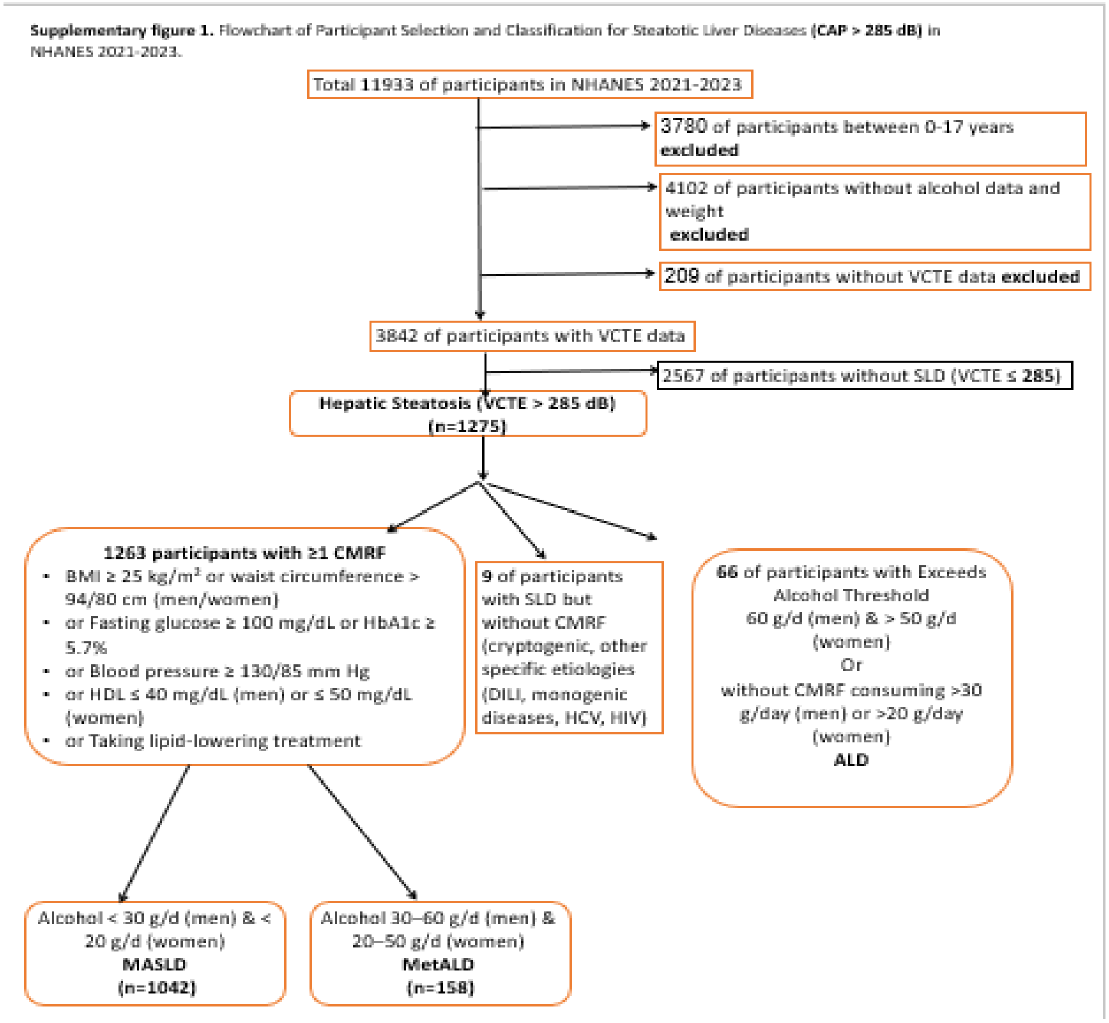

Supplementary Figure 2. Flowchart of Participant Selection and Classification for Steatotic Liver Disease (CAP > 285 dB) in NHANES 2017-2020.

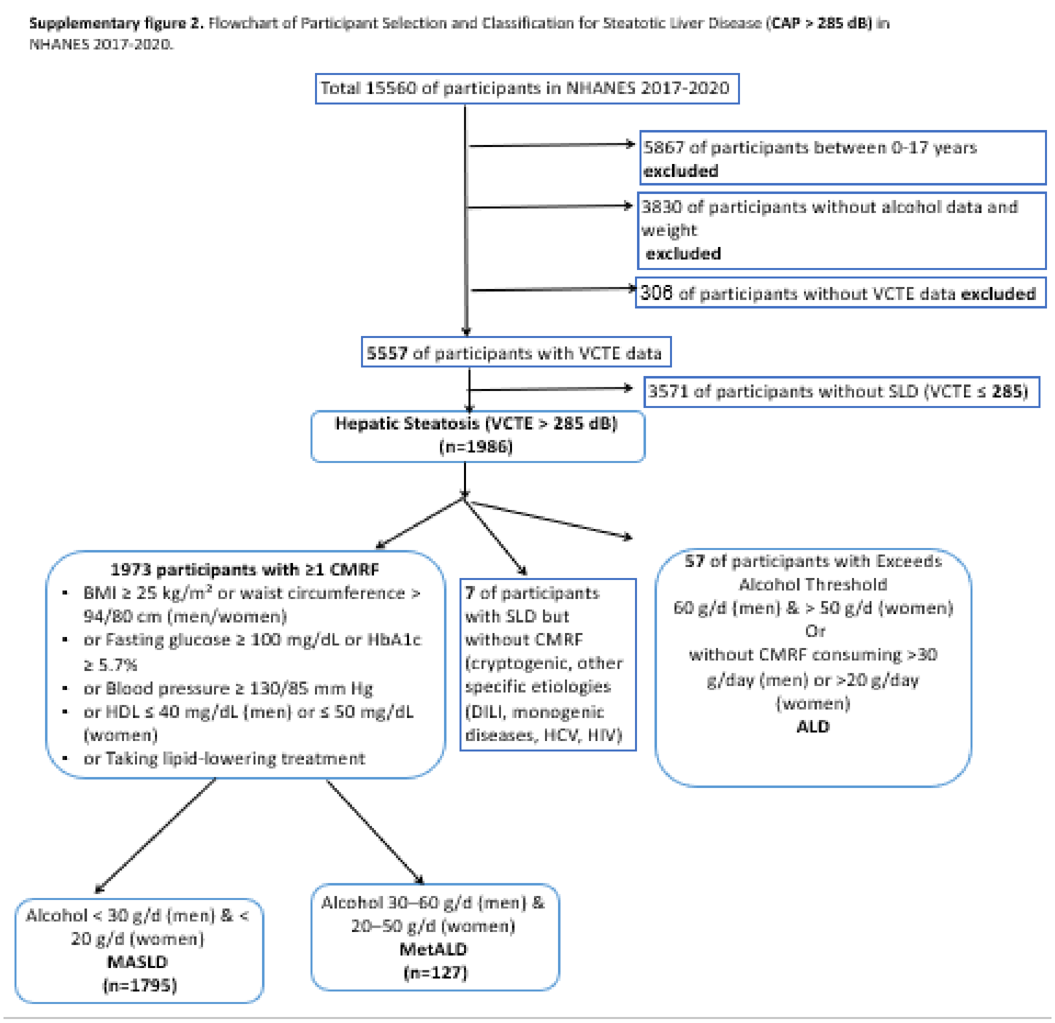

Supplementary Figure 3. Flowchart of Participant Selection and Classification for Steatotic Liver Disease (CAP ≥263 dB) in NHANES 2021-2023.

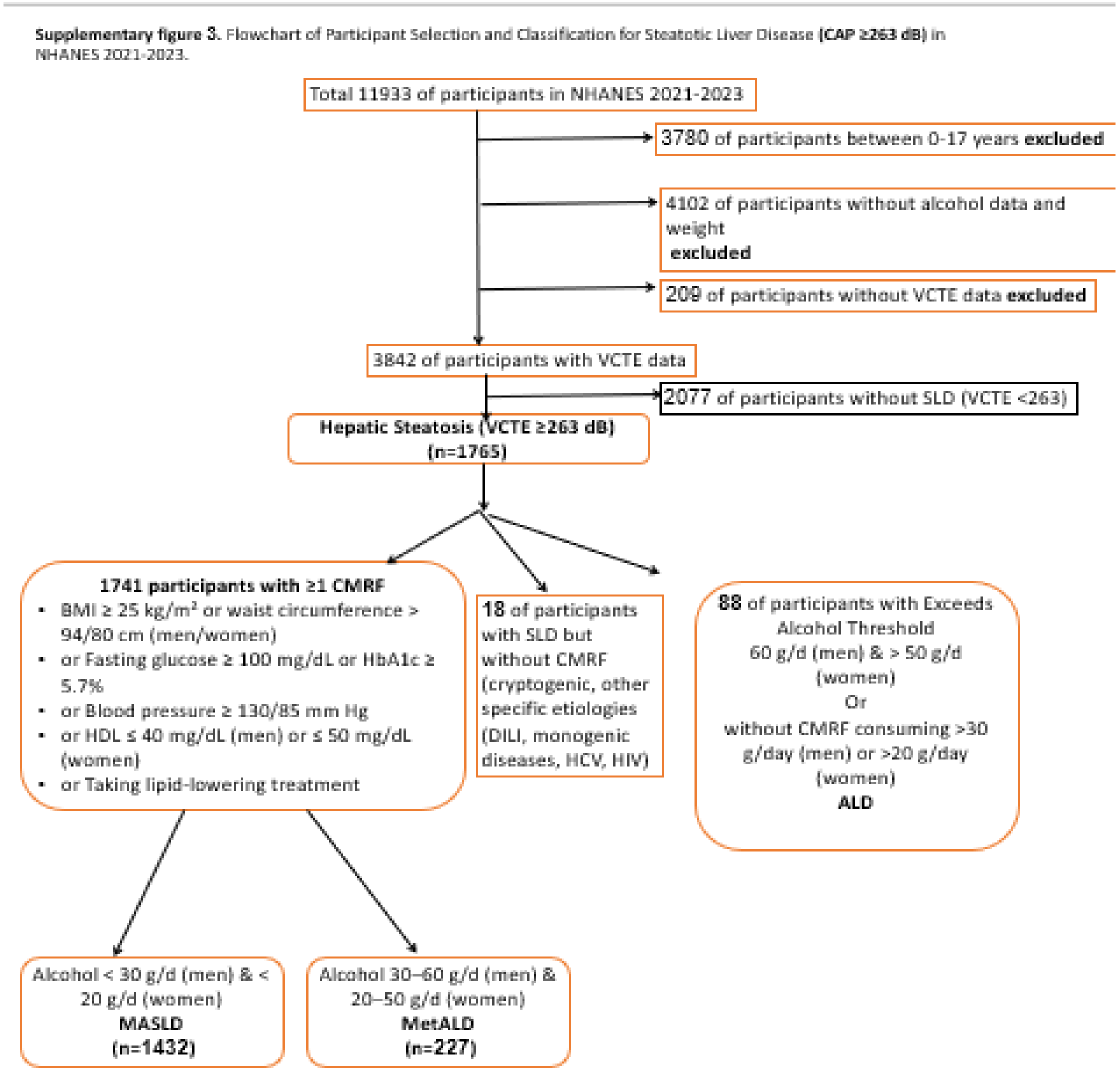

Supplementary Figure 4. Flowchart of Participant Selection and Classification for Steatotic Liver Disease (CAP ≥263 dB) in NHANES 2017-2020.

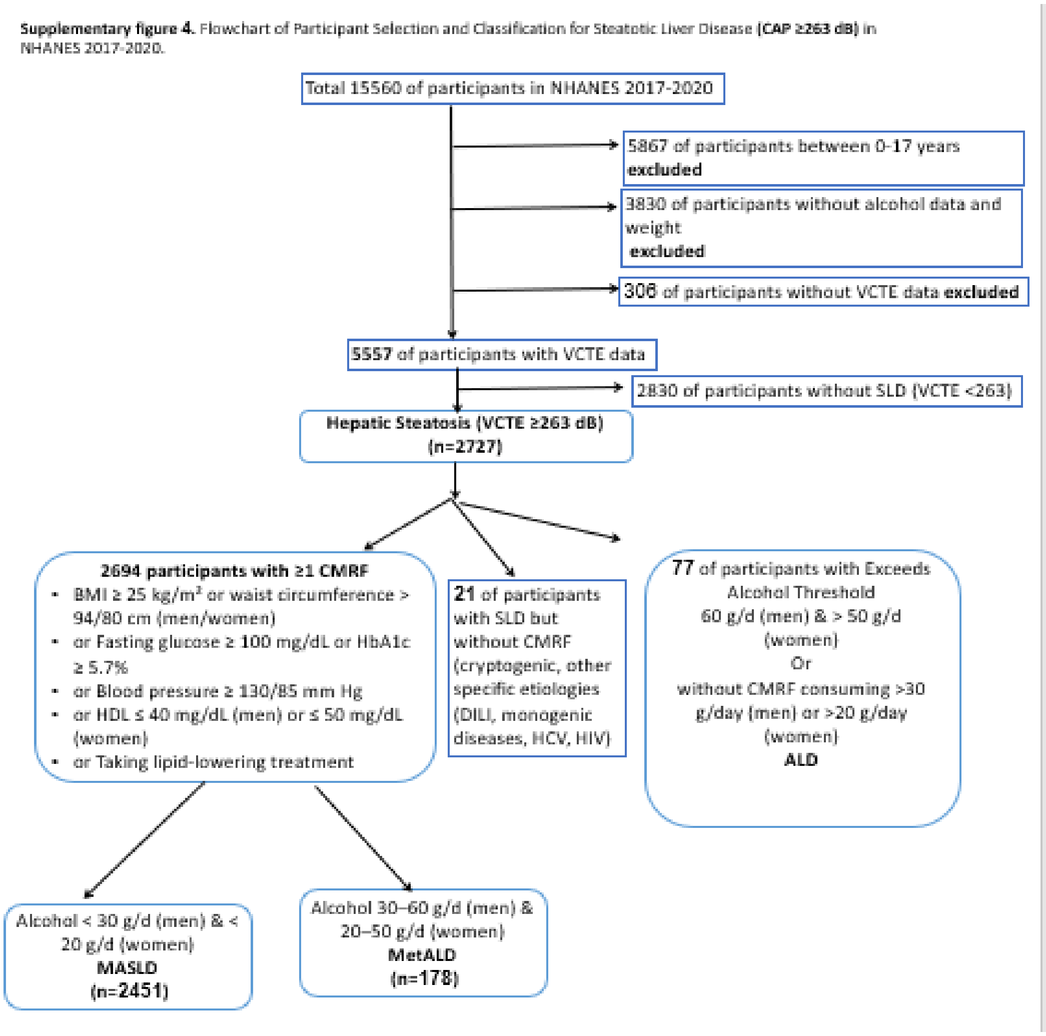

Supplementary Figure 5. Flowchart of Participant Selection and Classification for Steatotic Liver Disease (CAP > 248 dB) in NHANES 2021-2023.

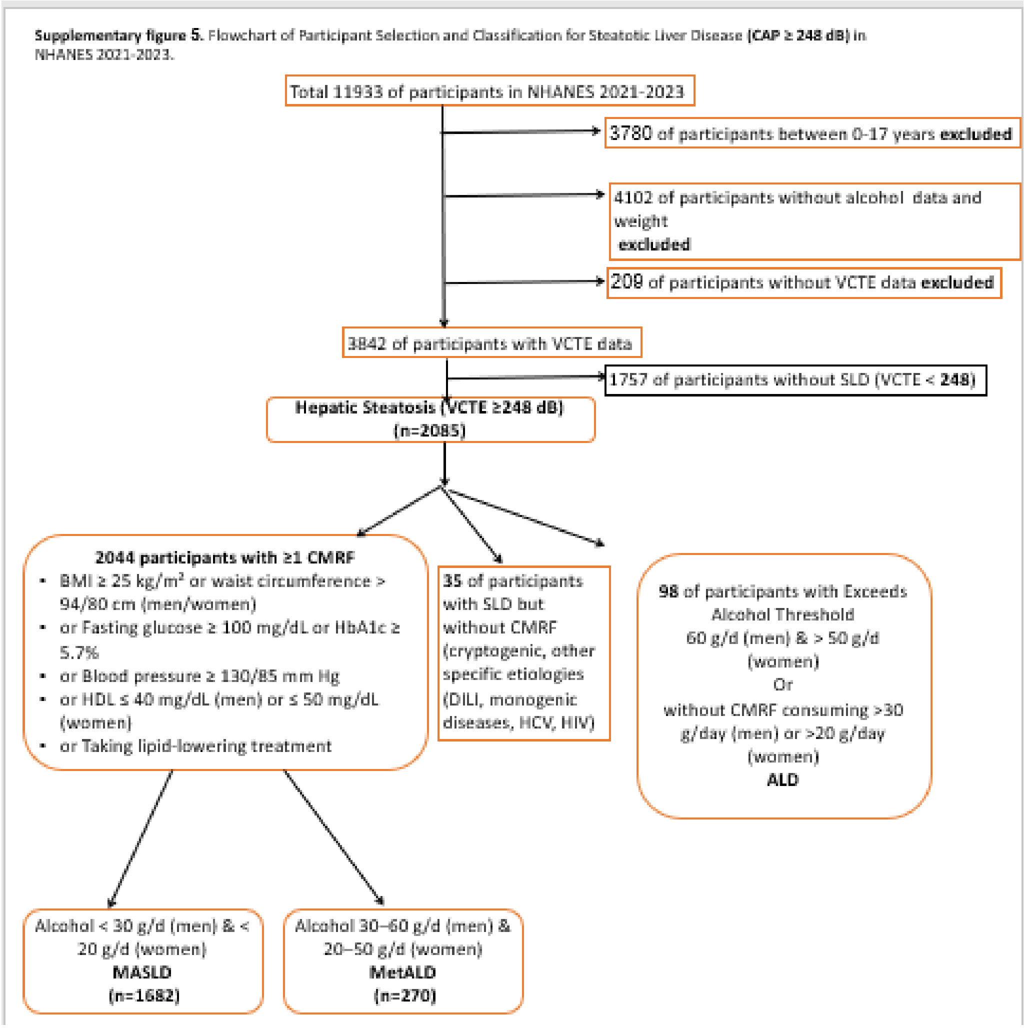

Supplementary Figure 6. Flowchart of Participant Selection and Classification for Steatotic Liver Disease (CAP ≥ 248 dB) in NHANES 2017-2020.

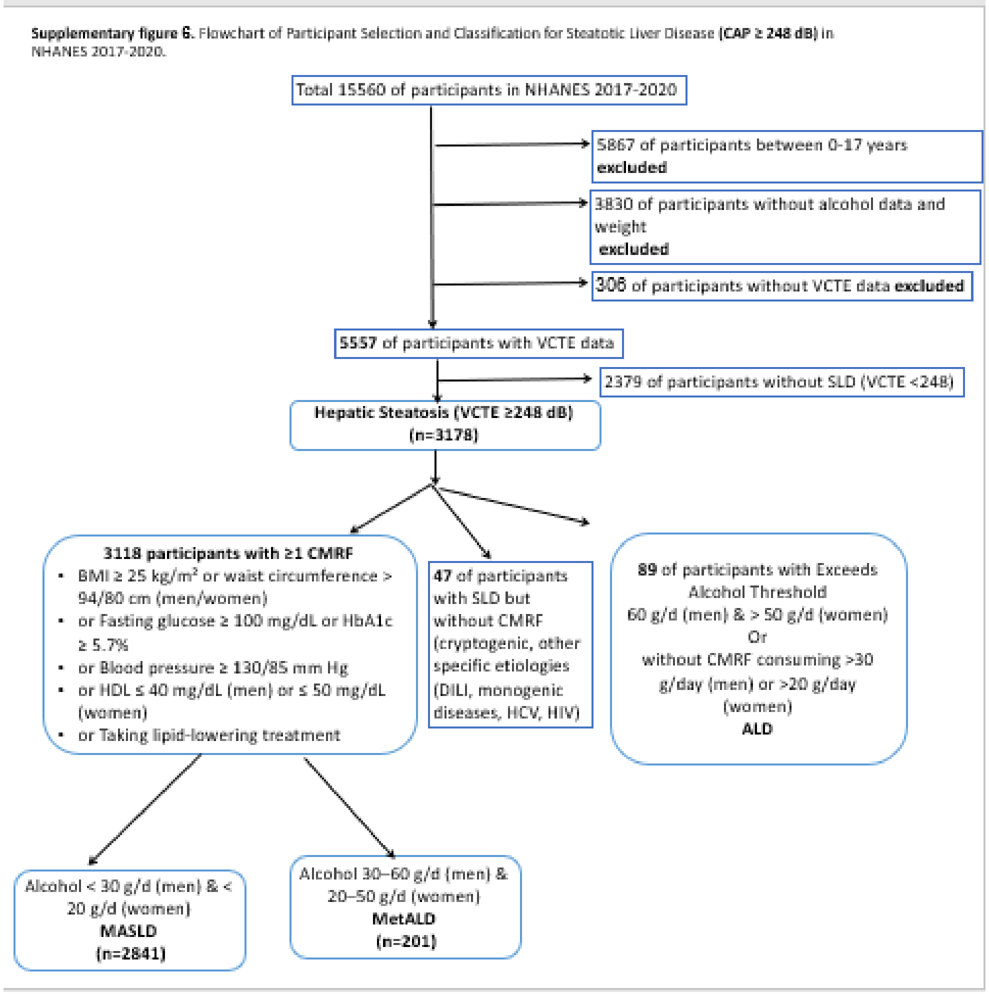

Supplementary Figure 7. Comparison of Age-Adjusted Mean Liver Stiffness Measurement (kPa) Among U.S. Population aged 18 Years or Older and Across Demographic Subgroups in Pre-Pandemic (2017–2020) and Pandemic (2021–2023) NHANES Cycles. **Panel A** displays the overall and gender-specific comparison (Female and Male); **Panel B** shows the racial/ethnic group comparison (Hispanic, Non-Hispanic Asian, Non-Hispanic Black, Non-Hispanic White, and Other); **Panel C** illustrates the socioeconomic group comparison (Higher Income, Middle Income, Near Poor, and Poor); and **Panel D** highlights the age group comparison (18-34, 35-49, and 50-64). Estimates are presented with error bars representing the standard error. Statistically significant differences between pre-pandemic and pandemic periods are indicated by an asterisk (*) with a p-value of ≤ 0.05.

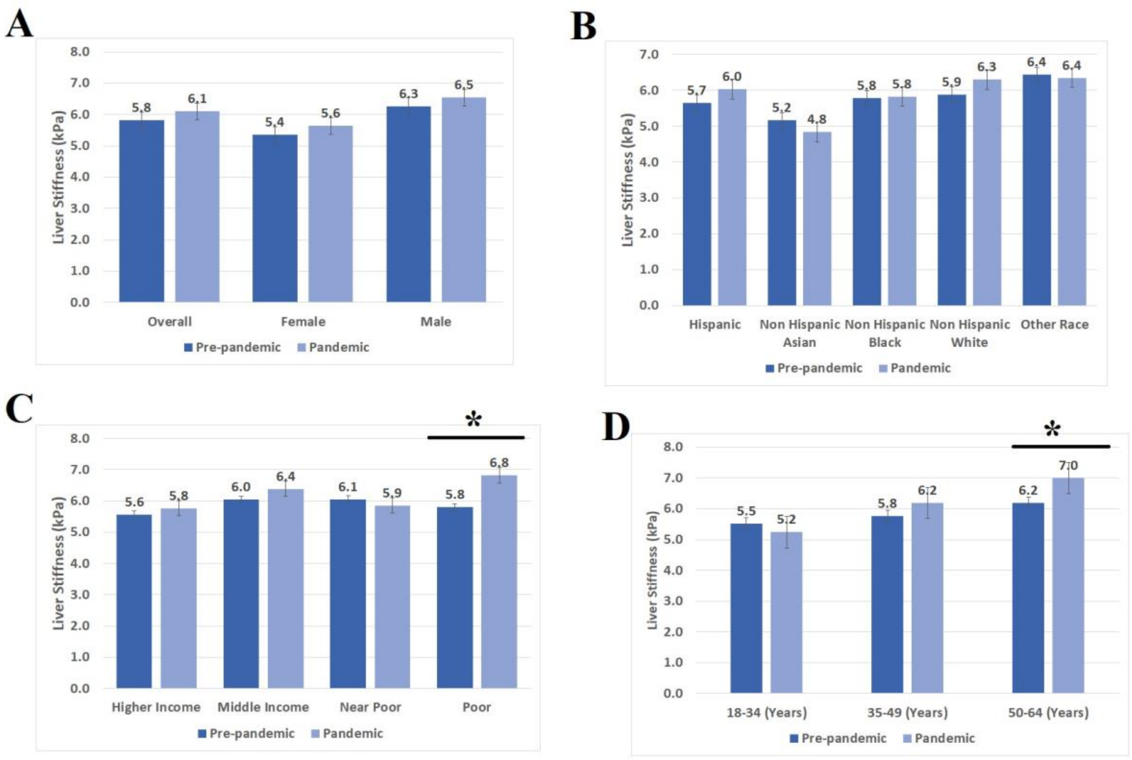

Supplement: Supplementary file 2 [file hc9-9-e0806-s002.pdf]
